# Mutated CYP17A1 promotes atherosclerosis and early-onset coronary artery disease

**DOI:** 10.1186/s12964-023-01061-z

**Published:** 2023-06-27

**Authors:** Ting-Ting Wu, Ying-Ying Zheng, Xiang Ma, Wen-Juan Xiu, Hai-Tao Yang, Xian-Geng Hou, Yi Yang, You Chen, Yi-Tong Ma, Xiang Xie

**Affiliations:** Department of Cardiology, First Affiliated Hospital of Xinjiang Medical University, No. 137, Liyushan Road, Urumqi, 830011 People’s Republic of China

**Keywords:** Early-onset coronary artery disease, CYP17A1, Pathophysiology, Atherosclerosis

## Abstract

**Background:**

Coronary artery disease (CAD) is a multi-factor complex trait and is heritable, especially in early-onset families. However, the genetic factors affecting the susceptibility of early-onset CAD are not fully characterized.

**Methods:**

In the present study, we identified a rare nonsense variant in the *CYP17A1 *gene from a Chinese Han family with CAD. To validate the effect of this variation on atherosclerosis and early-onset coronary artery disease, we conducted studies on population, cells, and mice.

**Results:**

The mutation precisely congregated with the clinical syndrome in all the affected family members and was absent in unaffected family members and unrelated controls. Similar to the human phenotype, the *CYP17A1*-deficient mice present the phenotype of metabolic syndrome with hypertension, increased serum glucose concentration, and presentation of central obesity and fatty liver. Furthermore, *CYP17A1* knockout mice or *CYP17A1* + *ApoE* double knockout mice developed more atherosclerotic lesions than wild type (WT) with high fat diary. In cell models, *CYP17A1* was found to be involved in glucose metabolism by increasing glucose intake and utilization, through activating IGF1/mTOR/HIF1-α signaling way, which was consistent in *CYP17A1* knockout mice with impaired glucose tolerance and insulin resistance.

**Conclusions:**

Through our study of cells, mice and humans, we identified *CYP17A1* as a key protein participating in the pathophysiology of the atherosclerotic process and the possible mechanism of *CYP17A1* C987X mutation induced atherosclerosis and early-onset CAD involving glucose homeostasis regulation was revealed.

Video Abstract

**Supplementary Information:**

The online version contains supplementary material available at 10.1186/s12964-023-01061-z.

## Introduction

Coronary artery disease (CAD) has a familial component. Moreover, the genetic contribution to CAD is more pronounced in premature forms of CAD [1, 2]. A reported family history (FH) of CAD had confirmed to be an independent risk factor for CAD event. The first-degree relatives with CAD present an elevated prevalence of metabolic abnormalities [3, 4], healthy relatives with a parental or sibling history of CAD demonstrated a higher burden of metabolic syndrome (Mets) [4, 5] and cardiovascular disease [6–8], including subclinical or clinic atherosclerosis [9–11], myocardial infarction(MI) [12–14], dyslipidemia [15], and showed defects in myocardial perfusion [16], endothelial dysfunction [17–19] and electrocardiographic abnormalities [20]. A variety of convergent observations suggest that independent genetic defects may be at work in familial forms of CAD [21–23]. Therefore, it is of great importance to identify genes that are susceptible to CAD. Whether causative genetic defects suffice to trigger the development of familial forms of CAD independently is a major issue that has profound implications for predictive and preventive medicine, as well as for research endeavors.

Recently, whole genome scans have been increasingly used and performed on a large number of patients with complex diseases such as CAD. By affected sibling-pair analyses, genome regions that were over shared between siblings were screened subsequently for specific sequence variations associated with the disease [24]. A specific sequence variant of CYP17A1 associated with CAD was found in our study through whole-exome sequencing and linkage analysis. The mechanisms underlying this familial clustering and the molecular genetic basis of CAD remain to be elucidated. Consequently, cell and mouse models have been studied to elucidate the pathways by which CYP17A1 is active in atherosclerotic processes.

## Materials and methods

Please see Additional file 1.

## Results

### Identification of a *CYP17A1* variant associated with early-onset CAD

**Fig. 1:**
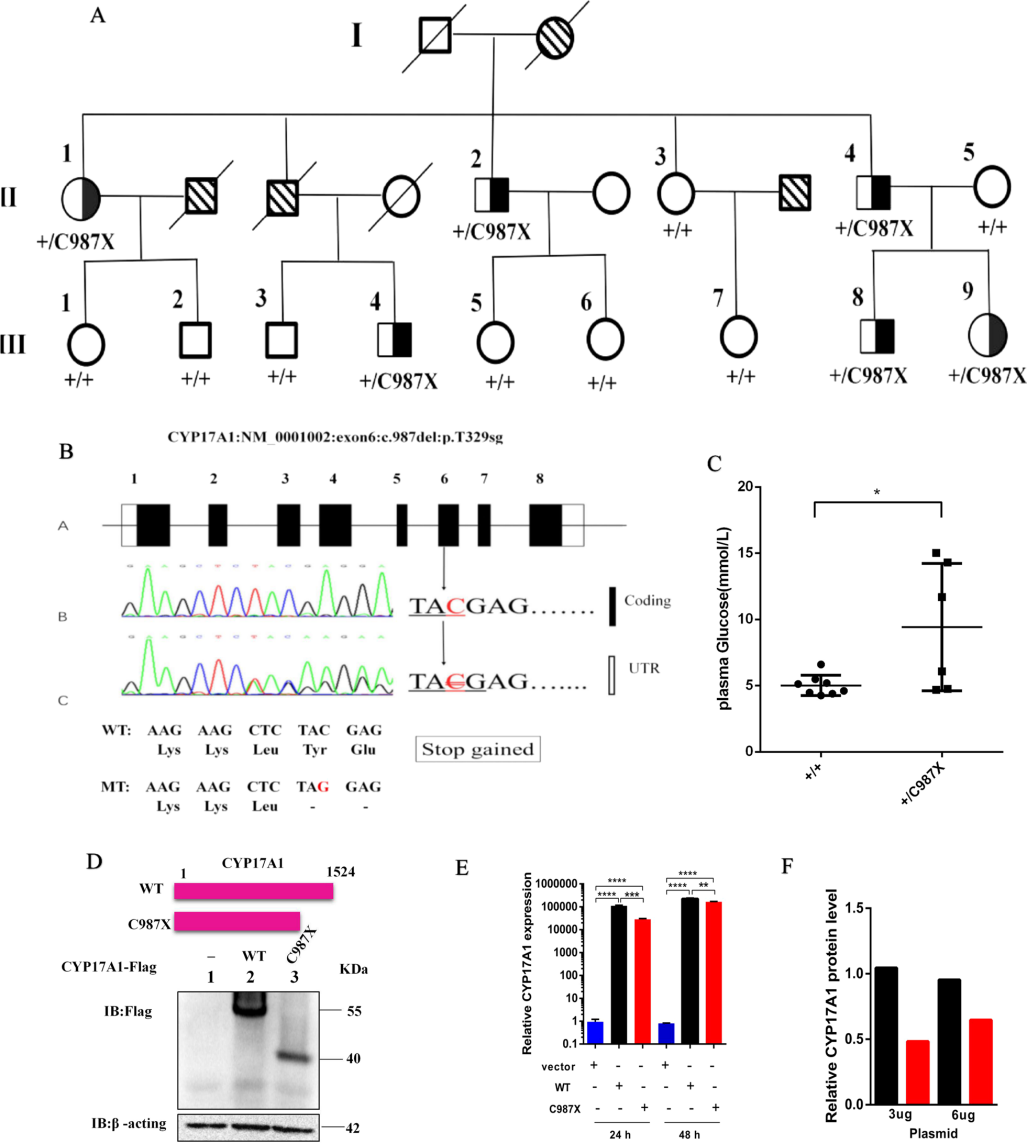
Identification of the C987X mutation (Tyr-to-stop-gain substitution at the amino acid site 329, a new truncation mutant, CYP17A1ΔECD: missing the N-terminal ectodomain) in the CYP17A1 gene from a Chinese Han family with inherited coronary artery disease (CAD). **A** Pedigree of a Chinese Han family with CAD. Squares and circles indicate males and females, respectively. Roman numerals indicate generations. Arabic numerals indicate individual family members. CYP17A1 genotype (half filled for C987X carriers, open for wild-type individuals) are shown below each square or circle. A shaded circle or square indicates that the family member had CAD alive, a slash on circle or square indicates that the member was died, and with a shadow and a slash simultaneously indicate that the family member died of CHD. **B** Genomic structure of human CYP17A1 gene. The del of cytosine in exon 6 causes a stop gain occurrence (indicated by arrow). DNA sequencing data of an unaffected man (III: 3) and an affected man (III: 4) with the heterozygous mutation in CYP17A1. **C** Glucose levels of the members of Family 1 (A). Data are expressed as mean ± SD. Statistical analyses, unpaired t test. *P < 0.05. **D** The protein levels of CYP17A1 and truncation mutant. Plasmids encoding Myc-tagged CYP17A1(WT) or CYP17A1(C987X) were transfected into HEK293T cells, and cells were harvested for Immunoblots analysis 48 hours later. β-acting was used as a loading control. **E** Relative mRNA levels of CYP17A1(WT) or CYP17A1(C987X) transfected at indicated time. **F**, Densitometric analysis of CYP17A1(WT) or CYP17A1(C987X) proteins shown in (**D**). Data are presented as mean±SD. Student’s *t* test; ns., no significant. ****P < 0.0001. *n*=3 biological replicates

In this study, we found a Chinese Han family (named Family 1) with inherited CAD (Fig. 1A). To identify CAD associated single nucleotide variant (SNV), all members (including 6 affected ones and 8 without clinical syndrome) in this family were analyzed by whole-exome sequencing. For data processing, using a dominant model, filtering against common variants, and considering functional prediction for the mutation, we narrowed potential candidates to a heterozygous deletion of the *CYP17A1* gene (*CYP17A1*: NM 0001002: exon 6: c.987del: p. T329sg, Tyr-to-stop-gain substitution at the amino acid site 329) on chromosome 10 (Additional file 1: Fig. S1). We then validated the variant by Sanger sequencing and confirmed this SNV to be the only one that congregated with the CAD phenotype within this family. As previous genome-wide association study (GWAS) had confirmed, the polymorphisms of *CYP17A1* were associated with hypertension [25, 26] and CAD [27], and successively be proved in Asian [28–30] and Caucasian [31, 32], also in our previous studies [33, 34]. Together these analyses suggest the *CYP17A1*-C987X SNV as a responsible variant for CAD in this family. The genomic structure of *CYP17A1* was shown in Fig. 1B. Detailed information about Family 1 is listed in Additional file 1: Table S1A. The plasma fasting blood glucose (FBG) levels of *CYP17A1*-C987X carriers (+/C987X) were significantly higher than those of wild type (WT) individuals (Fig. 1C). Due to the regular use of statins lipid-lowering drugs, the triglyceride (TG) significantly reduced in *CYP17A1*-C987X carriers, the plasma levels of total cholesterol (TC), high-density lipoprotein cholesterol (HDL-C), low-density lipoprotein cholesterol (LDL-C), urea nitrogen, creatinine and uric acid, were similar in both *CYP17A1*-C987X and WT individuals (Additional file 1: Table S1B). To our knowledge, the *CYP17A1*-C987X is a previously unknown mutation and has not been reported in any published databases. To determine whether this mutation is associated with CAD, it was then verified by direct sequencing in a case–control population (detailed information is listed in Additional file 1: Table S2). However, none C987X variant was found in a total of 1028 (576 CAD and 452 healthy control) individuals. We further sequenced the coding regions of *CYP17A*1 in 5 families and 150 sporadic CAD subjects. Neither C987X variant was found (Additional file 1: Fig. S2A). Nevertheless, a promoter region variation in *CYP17A1* (c.-14G > A) was identified in one family but not in 150 sporadic CAD subjects. The *CYP17A1* c. -14G > A mutation congregated with the CAD phenotype within this family (Additional file 1: Fig. S2B). It is well known that promoter alterations can significantly change the transcriptional activity of corresponding genes. The prediction of transcription factor binding site and CpG Island in the promoter regions was performed using proscan and CpG Island Search online prediction software. The presence of CpG islands was not detected, and the mutation was capable of contributing to the change of transcription factor binding sites (data not shown), which may affect the expression of *CYP17A1* gene, the activity of related enzymes, and may be related to the pathogenesis of CAD.

### The C987X substitution decreases CYP17A1 stability

**Fig. 2:**
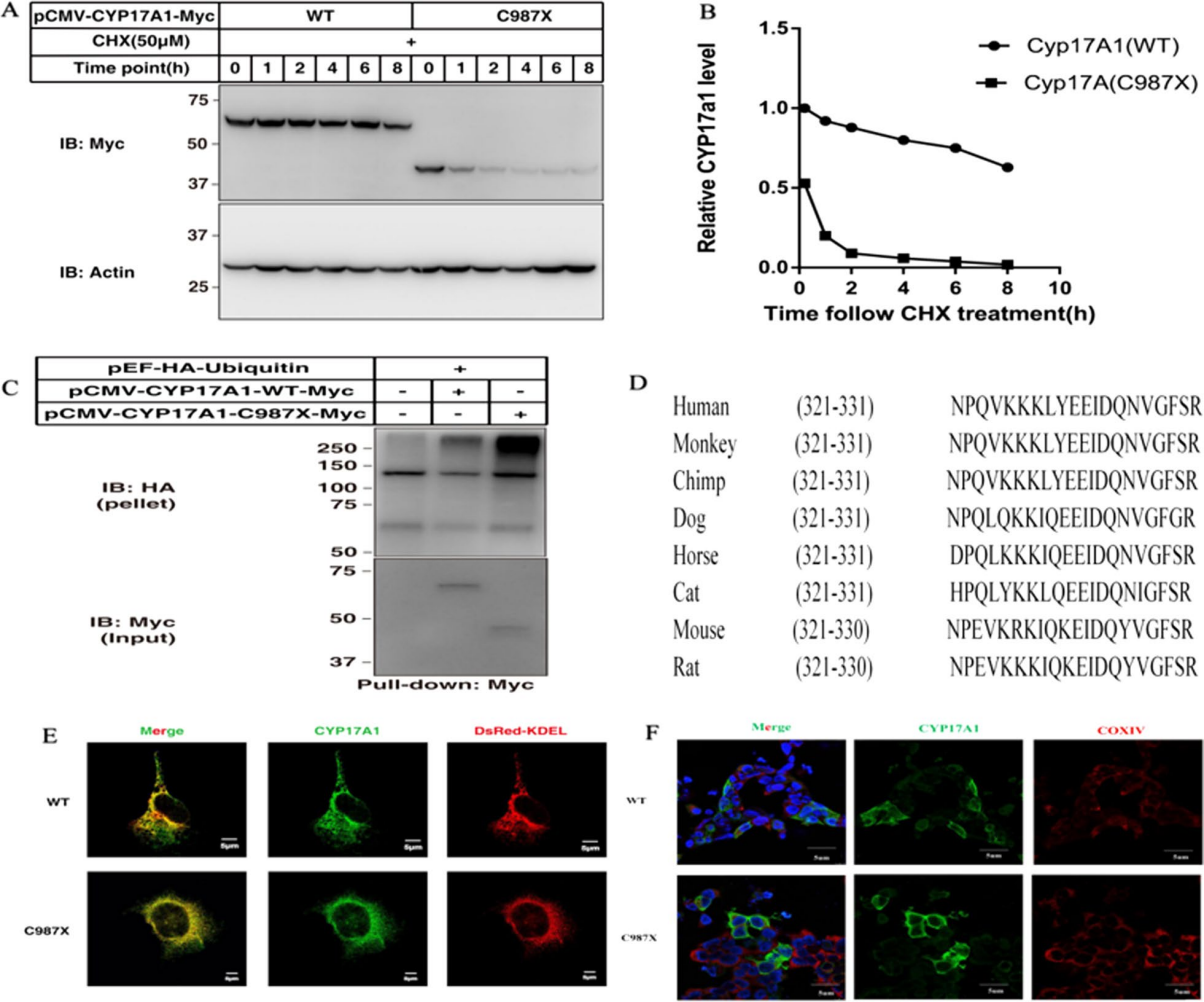
The C987X residue is critical for CYP17A1 stability. **A** HEK 293T cells were transfected with plasmids encoding CYP17A1(WT) or CYP17A1(C987X). After 48 hours, cells were treated with 100 μmol/L cycloheximide (CHX) for the indicated periods. **B** Densitometric analysis of CYP17A1(WT) and CYP17A1(C987X) proteins shown in (A). The densitometry of CYP17A1(WT) protein at 0 hour is defined as 1. **C** HEK293T cells were transfected with indicated plasmids and treated with 10 μm MG132 for 30 minutes. Cells were harvested, CYP17A1(WT) or CYP17A1(C987X) was immunoprecipitated by the anti-Myc coupled agarose and probed for the anti-HA antibody. Results shown are representative of two independent experiments. **D** Alignment of sequences flanking the C987X residue of CYP17A1 in various species. **E** Subcellular localization of CYP17A1(WT) or CYP17A1(C987X). HEK 293T cells were transfected with a plasmid encoding Myc-FLAG and stained with anti-FLAG and anti-KDEL antibodies. Scale bar =10 μm. **F** HEK293T cells were transfected with a plasmid encoding CYP17A1-FLAG and stained with anti-Myc tag and anti-COX IV antibodies, followed by microscopic analysis of CYP17A1 expression using a confocal microscope

We then examined the effect of C987X substitution on *CYP17A1* expression by transfecting HEK293T cells with a plasmid expressing human *CYP17A1* tagged with a N-terminal FLAG. Compared with the WT protein (about 55KD) form of CYP17A1, the C987X mutant produced a new truncation protein (about 40KD) (named CYP17A1∆ECD: missing the N-terminal ectodomain) which caused a 20% truncation of the CYP17A1 protein and exhibited lower protein levels (Fig. 1D, F). Transfecting with *CYP17A1*(WT) or *CYP17A1*(C987X), the mRNA abundance of *CYP17A1*were increased, regardless of the amounts or time transfected, *CYP17A1*(WT) is more expressive than *CYP17A1*(C987X) (Fig. 1E). The half-live of WT and C987X mutant were measured using a cycloheximide chase assay. The C987X mutant protein was degraded rapidly with a half-life of ≈ 1 h, whereas the *CYP17A1*(WT) was more stable with a half-life of ≈ 8 h (Fig. 2A, B). We next transfected HEK293T cells with WT and C987X, applied proteasome inhibitor MG132, the C987X mutation drastically increased CYP17A1 ubiquitination (Fig. 2C). The amino acid sequences flanking the C987X residue are relatively conserved across species (Fig. 2D). To confirm the subcellular location of *CYP17A1*(WT) and *CYP17A1* (C987X), we performed immunofluorescence experiments by co-staining transfected *CYP17A1*(WT) or *CYP17A1*(C987X) together with the endogenous ER marker KDEL or mitochondria marker COXIV. *CYP17A1*-FLAG was largely colocalized with KDEL (Fig. 2E), not in mitochondria (Fig. 2F), indicating that CYP17A1 is indeed an ER-localized protein. C987X is also co-located with ER, but not all of them, suggesting there may be a small amount of escape. Together, these results suggest that CYP17A1 is degraded through the ubiquitin–proteasome pathway, and the C987X mutation decreases protein stability.

### The CYP17A1 involved in glycometabolism

**Fig. 3:**
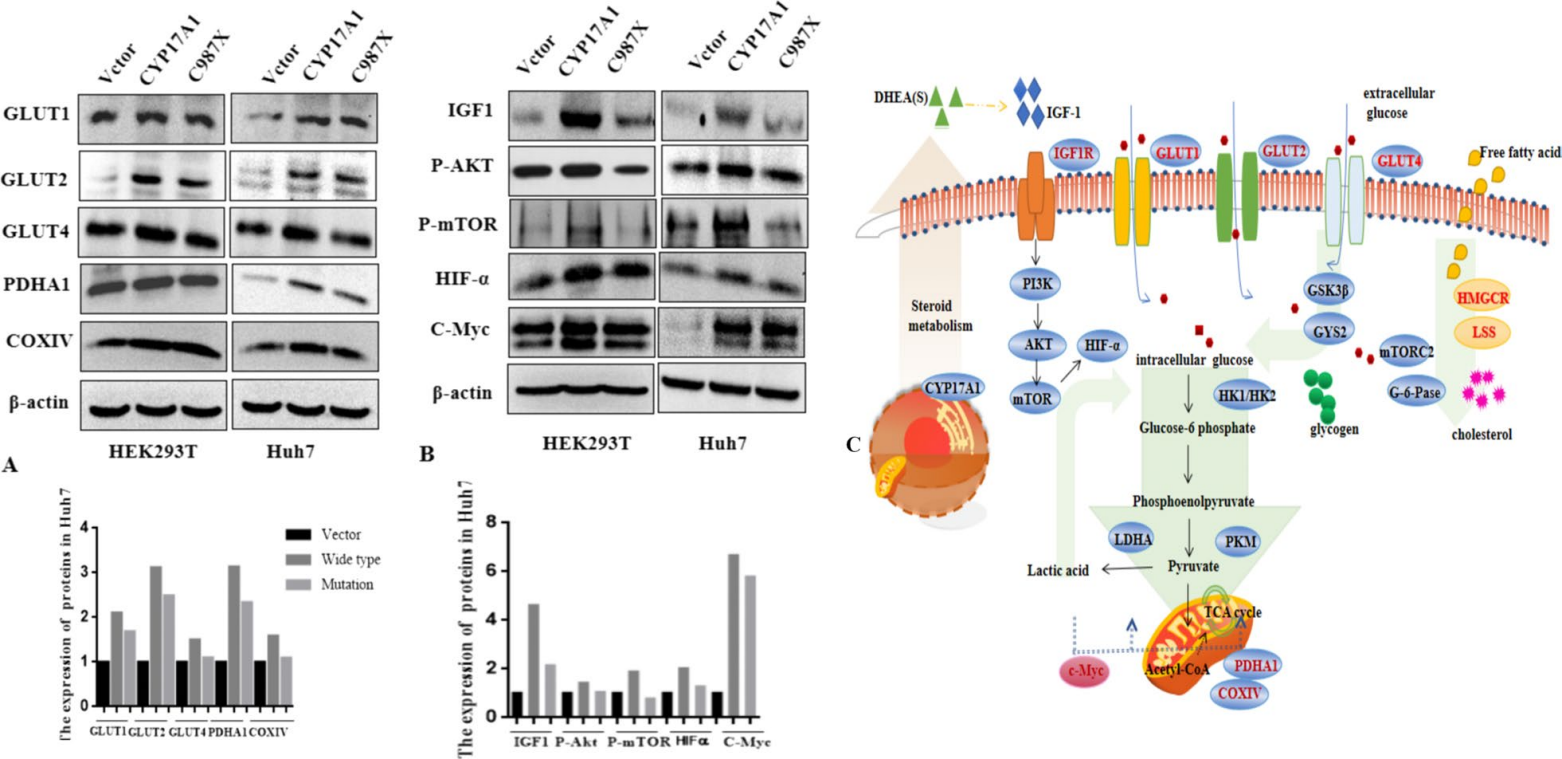
The expression profiles of key enzymes and regulators of glycometabolism in response to CYP17A1 over-expression. HEK293T cell or Huh 7 cells were transiently transfected with plasmids encoding CYP17A1(WT) or CYP17A1(C987X). After 48h following transfection, cell lysates were prepared and subjected to western blot analysis. β-actin expression was used as a loading control. **A** The effect of CYP17A1 and C987X mutation on key enzymes of glucose transport and aerobic oxidation in HEK293T or Huh7 cells. **B** The effect of CYP17A1 on regulators of glycolysis. **C** Schematic model of the CYP17A1-mediated glycometabolism

RNA-sequencing in HEK293T stable cells with C987X deletion constructed by CRISPR/Cas9 (Additional file 1: Table S3), together with Gene ontology (GO) and KEGG pathway analysis, we found that CYP17A1 may be related to glycolytic signaling pathways, then parts of key genes (Additional file 1: Table S4) were independently validated in stable or transient transfection cells by quantitative polymerase chain reaction (PCR), and found parts of them changed (Additional file 1: Fig. S3A). To investigate the expression profile in CYP17A1-induced glycometabolism, we analyzed the expression of rate limiting enzymes in transiently transfected cells with *CYP17A1*(WT) or *CYP17A1* (C987X) plasmid. It showed that over-expression CYP17A1, promoted the expression of the glucose transporter enzymes in both HEK239T and Huh7 cells (Fig. 3A) and the intracellular level of 2-NDBG was significantly increased (Additional file 1: Fig. S3B). These results may indicate that *CYP17A1* promoted the glucose uptake. Over-expression of *CYP17A1* significantly reduced the glucose level of medium but increased lactate excretion (Additional file 1: Fig. S3C, D). Further, we found the expression of IGF1-R (insulin-like growth factor 1 receptor) was increased, and IGF1/mTOR/HIF1-α pathway was activated in HEK293T and Huh7 cells (Fig. 3B). To determine whether *CYP17A1* may impact tricarboxylic acid cycle (TCA), the expression of PDHA1and COXIV were detected. Our data showed that *CYP17A1* up-regulated PDHA1 and COXIV expression in Huh7 cell, not in HEK293T (Fig. 3A), suggesting that intracellular acetyl-CoA might be potentially stimulated in a cell type dependent manner. HMGCR and LSS involved in cholesterol synthesis were increased in both HEK293T and Huh7 cell (Additional file 1: Fig. S3E). Schematic model of the *CYP17A1*-mediated glycometabolism was shown in Fig. 3C. Overall, our data indicate that *CYP17A1*can be manifested in different patterns of signaling events and *CYP17A1* could mediate glucose metabolism by increasing the uptake and utilization of glucose. However, the action is significantly reduced due to the instability of the mutant protein. CYP17A1 mutations are associated with a significant reduction in the amount and efficiency of glucose transporters.

Cell culture medium contains two main carbon sources: glucose and glutamine. To evaluate the relative contribution of these carbon sources to *CYP17A1*-mediated cell proliferation, CCK8 assay was performed in *CYP17A1* WT or C987X overexpressing cells cultured in either complete medium (G+/Gln+), glucose depleted medium (G−/Gln+) or glutamine depleted medium (G+/Gln−). Our results demonstrate that transient in HEK293T cells expression of *CYP17A1* WT or C987X can’t increase cell proliferation (Additional file 1: Fig. S3F). ATP assayed results showed that the lack of glucose significantly reduced ATP synthesis, indicating that glucose plays a more important role in cellular energy synthesis (Additional file 1: Fig. S3G). Compared to glutamine, glucose served as a major carbon source in HEK293T cells, which is manifested by a more significant impact on cell viability (Additional file 1: Fig. S3F), as well as protein synthesis (Additional file 1: Fig. S3H).

### Possible action way of *CYP17A1* or *CYP17A1*∆ECD

To reveal the possible action way of *CYP17A1* or *CYP17A1*∆ECD, we immunoprecipitation the *CYP17A1*-containing complex from transient transfection HEK293T cells and identified its binding proteins by tandem mass spectrometry (Additional file 1: Fig. S4A). 1029 genes were co-detected, 339 in WT and 740 in *CYP17A1*∆ECD were detected separately. The peptides expressed by *CYP17A1*∆ECD versus WT with more than five folds change were listed in Additional file 1: Table S5. The anti-*CYP17A1* immunoprecipitation (IP) and the anti-IgG IP were similarly performed, and the identified proteins served as nonspecific binders. The *CYP17A1*-binding candidates specifically identified from anti-*CYP17A1* IP of WT samples are listed (Additional file 1: Fig. S4B). Notably, a number of heat shock proteins (HSP) were found, which had been confirmed that steroid receptors can be phosphorylated, and then form complexes with HSP. Interestingly, leucine-rich PPR motif-containing protein (LRPPRC) and PKM, both related to glycometabolism, were among the CYP17A1-binding candidates. With a combination of various bioinformatics tools, analyzing together with RNA seq results, we found some functional process and related genes which may be the key signaling transduction during CYP17A1 associated glycometabolism (Additional file 1: Fig. S5A–F). Silver staining of IP suggests a specific protein (about 35 kD) between CYP17A1 and CYP17A1∆ECD (Additional file 1: Fig. S5G).

### Deficiency of CYP17A1 generate metabolic syndrome characterization

**Fig. 4:**
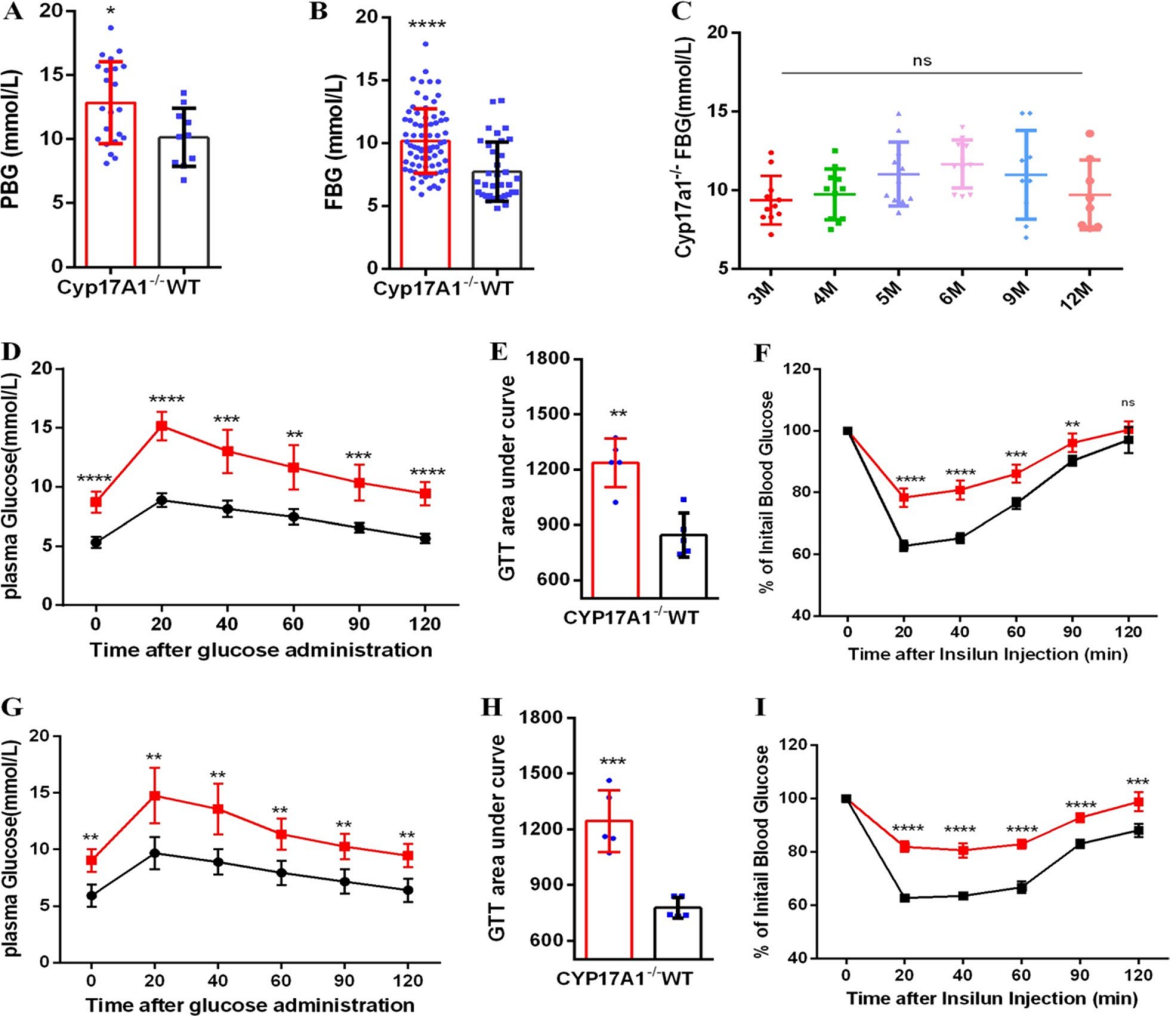
Glucose and insulin homeostasis in CYP17A1-/- mice. **A** postprandial blood glucose (PBG) levels of wild-type (n=10) and CYP17A1-/- mice (n=22). **B** fasting blood glucose (FBG) levels of wild-type (n=35) and CYP17A1-/- mice (n=75). Data are expressed as median with standard deviation. Nonparametric test. *P<0.05, **P<0.01, ***P<0.001, ****P<0.0001; **C** FBG levels of CYP17A1-/- mice at different months. One-way ANOVA. ns. indicates no significance. Glucose tolerance tests were performed in 6-mo-old (**D**), 12-mo-old (**G**) wild-type and CYP17A1-/- mice. Bar graphs denote calculated area under the curve for plasma glucose per hour of glucose administration,6-mo-old (**E**), 12-mo-old (**H**). Insulin tolerance tests were performed in 6-mo-old (**F**), 12-mo-old (**I**) wild-type and CYP17A1-/- mice. Insulin tolerance test data are presented as % of time at 0 plasma glucose levels

As previously reported [35], all homozygous mice produced by hybridization of heterozygous mice (created by TALEN) were infertile due to gonadal developmental defects, parts of them were Y chromosome-positive and the XY knockout mice had a female appearance (external genital phenotype) (Additional file 1: Fig. S6A), resembling the phenotype of human *CYP17A1* deficiency, leading to 46 XY differences/disorders of sex development. Whether feeding with HFD or chow, compared with WT mice, *CYP17A1*^−/−^ mice weight gain more significantly in time-dependent manner (Additional file 1: Fig. S7A) and show central obesity appearance (Additional file 1: Fig. S6A). *CYP17A1*^−/−^ mice significantly speed up hepatic steatosis or non-alcoholic fatty liver disease (NAFLD) (Additional file 1: Fig. S6B) with higher AST (Additional file 1: Fig. S7C) and ALT (Additional file 1: Fig. S7D), indicating by fat accumulation in the liver (Additional file 1: Fig. S8B). The amounts of white adipose tissue (WAT) adipocyte were also significantly increased in *CYP17A1*^−/−^ mice compared to WT (Additional file 1: Fig. S8A) with increased crown-like structures (indicator of inflammation) in the WAT (data not shown). The organ coefficients were calculated, in addition to the coefficient of visceral abdominal fat to weight significantly increased, the coefficients of liver, spleen, kidney, gonad to weight are significantly decreased in *CYP17A1*^−/−^ mice (Additional file 1: Fig. S7B). The systolic blood pressure of *CYP17A1*^−/−^ mice was significantly higher than that of WT (Additional file 1: Fig. S7E). *CYP17*A1^−/−^ mice with chow feeding at 6 or 12 months have increased plasma level of aldosterone, cortisol, resistin and insulin, decreased sex hormone level including dehydroepiandrosterone (DHEA), dehydroepiandrosterone sulfate (DHEAS), estrogens (E2), 17 hydroxyprogesterone (17-OHP), which were in consistent with *CYP17A1*^−/−^ mice HFD feeding at 6 or 9 months (Additional file 1: Fig. S9). Further, *CYP17A1*^−/−^ mice had higher fasting blood glucose (FBG) and postprandial blood glucose (PBG) than WT (Fig. 4A, B), the fluctuations of FBG in *CYP17A1*^−/−^ mice were independent of months (Fig. 4C). After an overnight fast, a statistically significant elevation in baseline blood glucose concentration was observed in 6-mo-old (Fig. 4D) and 12-mo-old (Fig. 4G) *CYP17A1*^−/−^ mice relative to WT mice. Consistent with loss of glycemic control, when administered a bolus of glucose, *CYP17A1*^−/−^ mice of all months showed elevated plasma glucose level, peaking at 20 min post-glucose administration (Fig. 4D, G). The area under the curve of the graph for plasma glucose vs. time was significantly increased in *CYP17A1*^*−*/−^ mice at 6 month (Fig. 4E), as well as12 month (Fig. 4H) of age compared with WT. In response to exogenous insulin administration, at 6 month, *CYP17A1*^−/−^ mice displayed a blunted plasma glucose response (Fig. 4F), which became more pronounced at 12 mo-old compared with WT mice (Fig. 4I). Together, these results above, all the *CYP17A1*^−/−^ mice present metabolic syndrome characterization including central obesity, as well as fatty liver, increased serum glucose concentration, insulin resistance and hypertension.

### Deficiency of *CYP17A1* in mice accelerate atherosclerotic plaque formation

**Fig. 5:**
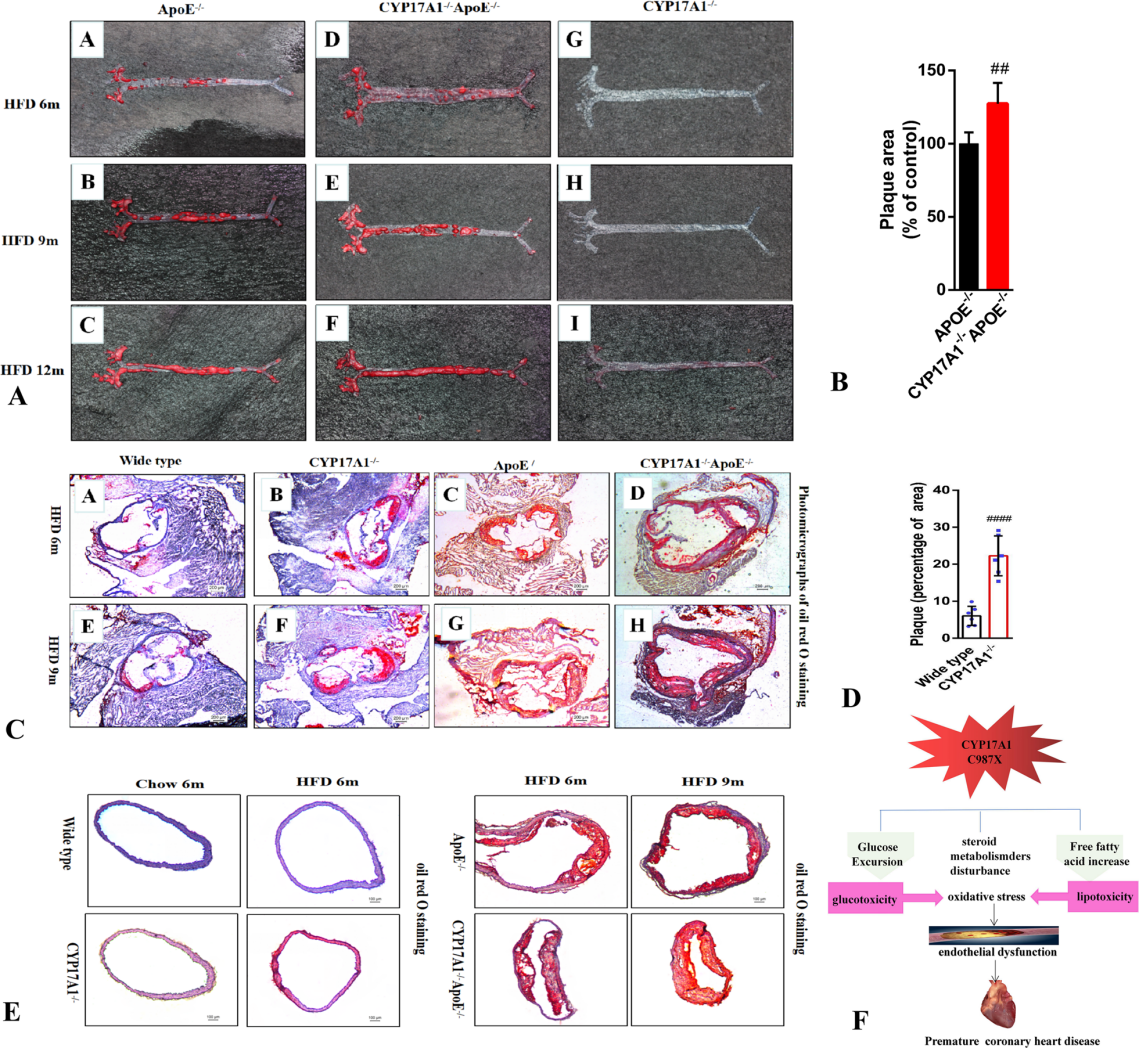
CYP17A1 whole-body knockout mice develop more aortic atherosclerotic plaques. **A** Representative images of Oil red stained atherosclerotic aortic plaques obtained from high fat diary (HFD)mice of different age. Genotype of knockout mice (left to right) ApoE-/-(ApoE konckout), CYP17A1-/-ApoE-/- (ApoE and CYP17A1 double knockout), CYP17A1-/- (CYP17A1 whole-body knockout) mice. Corresponding HFD time :6month (row 1),9month (row 2),12month (row 3). **B** Relative atherosclerotic aortic plaques area of ApoE-/-(n=6) and CYP17A1-/-ApoE-/- (n=6) mice. The plaques area of ApoE-/- is defined as 100%. Data are presented as mean±SD, unpaired t test, **P<0.01. **C** Atherosclerotic lesion histology in CYP17A1 knockout mice. Representative photomicrographs of Oil red O staining of cross-sections of aortic root in high fat diary WT, CYP17A1-/-, ApoE-/-and CYP17A1-/-ApoE-/- mice (from left to right) at 6 mo of age (**A**-**D**) and 9 mo of age (**E**-**H**). **D** Quantitative analysis of atherosclerotic lesion size in aortic root of CYP17-/- (n=6) mice and wide type(n=6). All histology images had the original magnification of 200μm. Data are presented as mean±SD, unpaired t test, ####*P*<0.0001. **E** Representative photomicrographs of Oil red O staining of cross-sections of aortic root vessel at 6-mo-old WT and CYP17A1-/- mice in chow or HFD diary mice. Photomicrographs of 6 and 9 mo-old ApoE-/-and CYP17A1-/-ApoE-/- mice with HFD. All histology images had the original magnification of 100μm. **F** Schematic model, the possible mechanisms of CYP17A1 C987X leading to CAD

In this study, we selected mice at 3, 6, 9 and 12 months for observation; however we didn’t find significant megascopic aortic plaque formation at 3 months in all genotypes (data not shown). In 6 or 9 or 12 months, a significant difference in plaque size was found, larger size of atherosclerotic plaques in *CYP17A1*^−/−^*ApoE*^−/−^ mice in comparison with control *ApoE*^−/−^ or *CYP17A1*^−/−^ mice feeding with HFD (with 0.25% cholesterol) (Fig. 5A). With time accumulation, the plaque area continued to increase, the plaque area differences between groups increased more pronounced, obvious advantages in *CYP17A1*^*−*/−^*ApoE*^−/−^ (Fig. 5B). Similarly, a cumulative increase was observed in cross-sections stain of aortic root in *CYP17A1*^−/−^*ApoE*^*−*/−^ comparison with *ApoE*^−/−^ (Fig. 5C). Although there none megascopic plaques in *CYP17A1*^−/−^ mice (Fig. 5A), surprised to find a significant plaque accumulation in the aortic root of *CYP17A1*^−/−^ compared to WT. The tendency to form plaques also present time dependent accumulation with significant (Fig. 5C, D). Furthermore, a significant lipid infiltration in the intima of vessels of *CYP17A1*^−/−^ compared to the WT with HFD, not observed in the chow (Fig. 5E), suggesting *CYP17A1* gene may interact with high-fat diet, which play together in the occurrence and development of atherosclerosis. Larger size of plaques in blood vessels section of *CYP17A1*^*−*/−^*ApoE*^−/−^ mice vs. *ApoE*^−/−^ (Fig. 5E). Together, these results demonstrate that ablation of *CYP17A1* accelerates vascular endothelial injury and promotes the formation of atherosclerosis in a time and diet–dependent manner.

As shown in Fig. 5F, we provided a schematic model of the mechanism of C987X mutation induced atherosclerosis and CAD.

## Discussion

For the first time, we identified a rare nonsense variant in *CYP17A1* as a disease mutation leads to familial early-onset CAD. Our data supply further expansion of genes that may predispose to early-onset CAD and also support further research into genetic determinants of cardiovascular risk. Then we conducted a detailed functional study of *CYP17A1* and its mutation in human, cells and animals. *CYP17A1* was found to be involved in glucose metabolism by increasing glucose intake and utilization, though activating IGF1/mTOR/HIF1-α signaling way. Due to the unstable of C987X mutation and its impaired function, it caused the loss of original function. In consistent with cellular results, *CYP17A1* knockout mice, apparent impaired glucose tolerance, insulin resistance, corresponding changes in the levels of hormones, accelerated vascular endothelial injury and promoted formation of atherosclerosis. Taken these together, our results suggest that *CYP17A1* may also be involved in glucose metabolism, in addition to the classical steroid regulation. When *CYP17A1* is mutated or functional impaired, it may be involved in the occurrence and development of coronary atherosclerosis through a variety of ways.

It has been shown that IGF1 has insulin-like metabolic effects as an essential regulator of cell growth, differentiation and apoptosis, which are regulated by a variety of factors including hormones, nutritional status and genetics. It plays a crucial role in the pathophysiological processes of cardiovascular diseases, endocrine and metabolic diseases, and tumors [36]. Recent studies have proved that IGF1 has a variety of metabolic and vascular protective effects, it can regulate insulin metabolism through phosphatidylinositol 3 kinase (PI3K)/protein kinase B(AKT) signaling pathway and related to blood lipid, which plays an important role in the occurrence and development of anti-atherosclerosis [37, 38]. In our study, we found that CYP17A1 could significantly up-regulate IGF1 expression and its downstream mTOR/HIF1-α signaling pathway. It has been reported that HIF1-α regulates glucose metabolism by inducing the expression of enzymes involved in the glycolytic pathway and glucose transporters (GLUTS) [39]. In agreement with our results, we find that CYP17A1 mutations are associated with a significant reduction in the amount and efficiency of glucose transporters, leading to glucose metabolism disorders. As blood glucose increases, on the one hand, the activity of glucose transporters is weakened, leading to a defective operation of the transport system. It can directly impact myocardial glucose uptake and utilization by reducing glucose transport through the membrane. Insulin deficiency, on the other hand, hinders the translocation and aggregation of glucose transporters, resulting in a significant reduction in the number of glucose transporters that can function. As the quantity and quality of glucose transporters is reduced, this can lead to an imbalance in the energy metabolism of cardiomyocytes and ultimately to impaired cardiac function [40].

Early-onset CAD (before 55 years in men and 60 in women) is more likely to reflect a genetic predisposition. Because of shared lifestyle risk factors and genetic predisposition, compared with the general population, siblings have at least double the risk, offspring and partners are also at increased risk [41, 42]. As relatives have an increased prevalence of modifiable risk factors, the first-degree relatives of patients admitted for early-onset CAD should be identified and then offered screening and treatment for risk factors. Moreover, people with the highest burden of genetic risk derived the largest relative and absolute clinical benefit from therapy [43]. Independent genetic defects may play an important role in familial early-onset CAD, which were confirmed by Mani et al. [21] and Keramati et al. [22] subsequently. Previous studies suggested the possible mechanism of early-onset CAD may includ an increased susceptibility to atherosclerosis [18], an increased tendency for thrombosis [44], proinflammatory responses [45], and subclinical atherosclerosis [9]. However, the mechanism is not entirely clear yet. *CYP17A1*, cytochrome P450 family 17 subfamily A member 1, is also named as *CPT7*, *CYP17*, *P450C17*, and *S17AH*. It is a cytochrome P450 monooxygenase encoding 17α-hydroxylase, 17, 20-lyase, involved in drug metabolism, synthesis of cholesterol, steroids, and other lipids and participating in the process of carbon–carbon bond scission [46]. Published evidence indicates that steroid hormones have a critical role in CAD and its imbalance could be a traditional risk factor for CAD. Estrogens appear to be protective in women [47, 48], while lower testosterone levels and dehydroepiandrosterone sulfate (DHEAS) have been linked to increased risk for CAD [49–52]. As *CYP17A1* is a key microsomal enzyme that converts pregnenolone and progesterone to their major downstream products: DHEAS, cortisol, testosterone, and estradiol [53]. It’s may relate to CAD for an imbalance in the level of sex hormones under some certain circumstances. The animal plasma steroid analyses revealed the hypothesis completely that *CYP17A1* may related to atherosclerosis by participating in the regulation of steroid. HMGCR and LSS over express with the transfection of *CYP17A1* plasmid in cells, strongly evidenced that *CYP17A1* may related to cholesterol metabolism. Aherrahrou et al. also found the increased atherosclerosis in *CYP17A1* XY knockout mice lacking testosterone associated with altered lipid profiles [35]. The relationship of *CYP17A1* with glucose metabolism has not been clarified clearly to date. In this context, we generated *CYP17A1*-deficient mice and cells models to discuss its impact on glucose metabolism. Lu et al. found *CYP17A1*/17-hydroxyprogesterone/ glucocorticoid receptor dependent pathway in the liver that mediates obesity-induced hyperglycemia [54]. However, we found over express *CYP17A1* gene in Huh7 cell could regulating glucose metabolism by increasing glucose intake, promoting glucose utilization, and improving insulin resistance by IGF1 signaling way, and we confirmed the expected disorder of glucose metabolism in *CYP17A1*-deficient mice with impaired glucose tolerance and insulin resistance. As for the difference of conclusion, we think it may be caused by the difference of background mice. Relevant research results are obtained by building 2 common models of severe obesity (leptin receptor deficient db/db mice and leptin deficient ob/ob mice), whereas, our background mice were C57BL/6. At the same time, *CYP17A1* is mainly produced in adrenal glands and sexual tissues in mice, while Lu et al. interfered with the expression of *CYP17A1* gene in the liver. What we performed was the complete knockout mice of *CYP17A1* gene. Therefore, we believe that *CYP17A1* at the systemic level has another way action of blood glucose regulation. As reported, the atherosclerosis was enhanced in castrated female [55, 56], the atherosclerosis development was detected in our female mice. It is conceivable that when *CYP17A1* is mutated or functional impaired, it may cause long-term fluctuation of blood glucose and increase the level of free lipids, which can aggravate lipid toxicity and glucose toxicity, further increase oxidative stress, cause endothelial dysfunction.

Then the corticosterone release was stimulated by inflammatory cytokines and nutritional overflow, which in turn accelerates the development of atherosclerotic changes. The result of co-IP suggests the possible interacting proteins and provides a new direction for our further study to reveal the possible mechanism of *CYP17A1* involvement in atherosclerotic and it also gives a new sight to the possible function of CYP17A1∆ECD protein.

### Limitations

Given that *CYP17A1* is also associated with essential hypertension and involved in corticoid and androgen biosynthesis, we failed to measure the relative endogenous hormone levels of Family 1 members.A limited number of patients with early-onset CAD were collected, and none C987X mutations were detected. It may helpful to understand the physiological function of CYP17A1 gene, by examining the expression of genes or proteins related to glucose metabolism in the CYP17A1 knockout mice. However, our animal model, which was a whole-body knockout of CYP17A1 gene, could not well simulate the mechanism of human CYP17A1 and its variants,its another limitation of our study. In vivo regulatory pathways may be more complex. It remains unclear whether alternative pathways are involved in the regulation of blood glucose and targeted knockout animal models are needed to better mimic the real situation in vivo and reveal its more precise regulatory mechanisms.

Given differences between mice and humans, functional studies through more clinical samples may be of more clinical value.

## Conclusion

These findings indicate a role for CYP17A1 in glucose homeostasis and associate its altered function with an inherited form of the early-onset CAD.

## Supplementary Information

**Additional file 1: Figure S1. **Schematic of whole-exome sequencing data processing and variant identification. **Figure S2.** The c.-14G>A variant in CYP17A1. (A) Schematic of identification CYP17A1 as a candidate gene for CAD susceptibility. (B) Pedigrees of the family 2 with c.-14G>A mutation. CYP17A1 genotype (half filled for c.-14G>A carriers, open for wild-type individuals) are shown below each square or circle. **Figure S3.** CYP17A1 over-expression involved in glycometabolism. (A) Relative mRNA levels of genes related to glycolytic pathway (screened by RNA-seq) in stably expressing C987X mutation or in HEK293T cells with over-expressed CYP17A1 WT or C987X.Vector as control. (B) CYP17A1 promotes the glucose uptake in HEK293T cells. HEK293T cells transfected with plasmids were labeled with intracellular level of 2-NBDG and subjected to measurement of flow cytometry. HEK293T cells cultured in a 6-well culture plate were transiently transfected with 3μg/well of plasmids encoding CYP17A1(WT) or CYP17A1(C987X). After 48h transfection, supernatants were harvested and subjected to the detection of glucose(C) or lactate(D) using assay kits. (E) After 48h transfection, cell lysates were prepared and subjected to western blot analysis. β-actin expression was used as a loading control. The effect of CYP17A1 and C987X mutation on key enzymes of cholesterol synthase in HEK293T and Huh7 cells were detected. (F) The influence of glucose and glutamine on CYP17A1-mediated cell proliferation. HEK293T cells with transient CYP17A1 WT or C987X expression were cultured in three different media: the complete medium with both glucose (Glu+) and glutamine (Gln+); the culture medium with glucose (Glu+) but without glutamine (Gln-); the culture medium without glucose (Glu-) but with glutamine (Gln+). After 24h incubation, cell proliferation in each culture condition was evaluated by CCK8 assay. (G)ATP level was measured by an assay kit. Data are presented as mean ± SD from at least three independent experiments. (H) The expression of CYP17A1 protein in different media, detection by WB. n=3 biological repetition. *, *p* < 0.0.5; **, *p* < 0.01; ***, *p* < 0.005, ****, *p* < 0.001.n.s., no significance. **Figure S4.** Identification of CYP17A1-binding proteins. (A)Schematic of the work flow of immunoprecipitation (COIP) followed by tandem mass spectrometry (MS/MS). (B) The top twenty candidate proteins that specifically identified in anti-CYP17A1 IP from HEK293T cell transfected with WT plasmids sample shown in (A). **Figure S5.** Identification of CYP17A1 functional paths. (A) Schematic of the work flow of combine analysis of CO-IP and RNA-seq. (B) Parts of candidate gene that specifically related to functional paths shown in (A). (C-F) Functional path clusters (the color represents the difference, the deeper the more significant) and interaction network (the color of different nodes represents different clusters, the connection line represents the genetic similarity between terms, and the size of nodes represents the number of genes enriched) in differences unique to wild-type (C and D) or to C987X truncation mutant (E and F). (G)Silver staining of IP. Specific protein (about 35 kD) between CYP17A1 and CYP17A1ΔECD were pointed with an arrow. **Figure S6.** Diagram of CYP17A1-/-mice. Upper part: (A) Representative photos of of appearance(A) in chow fed CYP17A1-/- heterozygote, homozygous and WT mice at 12 mo of age and relative diagram of the anatomy (B), 9 month of HFD appearance(C) of WT, CYP17A1-/- and CYP17A1-/-ApoE-/- mice. Lower part: Abdominal, epicardial and liver necropsy photos of representative 12-mo-old CYP17A1-/- heterozygote, homozygous and WT mice, showing increased adiposity in CYP17A1-/- vs. WT mice. Livers of CYP17A1-/- mice tend to display increased fat accumulation displaying yellow coloration compared with WT (F vs. C). **Figure S7.** Characterization of CYP17A1 whole-body knockout mice. (A)WT, CYP17A1-/- mice (n=8) were fed on chow diet or HCD (0.25% cholesterol). Weights were measured at 3,6,9,12 month. Data is expressed as mean ± SD. Statistical analyses, two-way ANOVA. (B)Tissues taken from 12-mo-old WT and CYP17A1-/- mice were immediately weighted by micro balance. Organ coefficient was calculated. Biochemical detector to detect serum AST(C) and ALT(D). (E)Blood pressures were measured indirectly by tail arteries in awake mice. Data are expressed as median with SD. Nonparametric test. #*P*<0.05, ##*P*<0.01, ###*P*<0.001, ####P<0.0001, compared with WT mice fed with HCD diet. *P<0.05, **P<0.01, ***P<0.001, ****P<0.0001, compared with WT mice fed with chow diet. ns, not statistically significant compared with WT mice fed with chow diet. NS, not statistically significant compared with WT mice fed with HCD. **Figure S8.** Adipose and liver histology in CYP17A1-/- mice. Upper part: Hematoxylin and eosin (H&E) staining of gonadal adipose sections from WT (A), CYP17A1-/-(B) mice at 12 mo of age. Lower part: H&E staining of liver sections. H&E staining of liver sections from chow fed female CYP17A1-/- mice at 3, 6,9 and 12 mo-old (A-D). WT female mice at 3, 6, 9and 12 mo(E-H), respectively. All histology images are X40 magnification; scale bar in bottom right =50μm. **Figure S9.** Metabolic characteristics of CYP17A1 whole-body knockout mice. WT and CYP17A1-/- mice at 6 or 12mo-old (n=6, each month/each genotype) with chow diet, WT and CYP17A1-/- mice a t6 or 9 mo-old (n=6, each month/each genotype with HCD. Plasma DHEA, DHEAS, plasma Estrogen, plasma aldosterone, plasma cortisol, plasma insulin, plasma resistin and plasma 17-hydroxyprogesterone were measured. Data are expressed as mean ± SD. Statistical analyses, two-way ANOVA. #*P*<0.05, ##*P*<0.01, ###*P*<0.001, ####*P*<0.0001, compared with WT mice fed with HCD diet. *P<0.05, **P<0.01, ***P<0.001, ****P<0.0001, compared with WT mice fed with chow diet.

## Data Availability

Not applicable.
