# Supplementary material for: Mutated CYP17A1 promotes atherosclerosis and early-onset coronary artery disease
Source: Cell Commun Signal. 2023 Jun 27;21:155. doi: 10.1186/s12964-023-01061-z (PMC10294473; doi:10.1186/s12964-023-01061-z)
Supplement: Supplementary file 2 — Additional file 1: Figure S1. Schematic of whole-exome sequencing data processing and variant identification. Figure S2. The c.-14G>A variant in CYP17A1. (A) Schematic of identification CYP17A1 as a candidate gene for CAD susceptibility. (B) Pedigrees of the family 2 with c.-14G>A mutation. CYP17A1 genotype (half filled for c.-14G>A carriers, open for wild-type individuals) are shown below each square or circle. Figure S3. CYP17A1 over-expression involved in glycometabolism. (A) Relative mRNA levels of genes related to glycolytic pathway (screened by RNA-seq) in stably expressing C987X mutation or in HEK293T cells with over-expressed CYP17A1 WT or C987X.Vector as control. (B) CYP17A1 promotes the glucose uptake in HEK293T cells. HEK293T cells transfected with plasmids were labeled with intracellular level of 2-NBDG and subjected to measurement of flow cytometry. HEK293T cells cultured in a 6-well culture plate were transiently transfected with 3μg/well of plasmids encoding CYP17A1(WT) or CYP17A1(C987X). After 48h transfection, supernatants were harvested and subjected to the detection of glucose(C) or lactate(D) using assay kits. (E) After 48h transfection, cell lysates were prepared and subjected to western blot analysis. β-actin expression was used as a loading control. The effect of CYP17A1 and C987X mutation on key enzymes of cholesterol synthase in HEK293T and Huh7 cells were detected. (F) The influence of glucose and glutamine on CYP17A1-mediated cell proliferation. HEK293T cells with transient CYP17A1 WT or C987X expression were cultured in three different media: the complete medium with both glucose (Glu+) and glutamine (Gln+); the culture medium with glucose (Glu+) but without glutamine (Gln-); the culture medium without glucose (Glu-) but with glutamine (Gln+). After 24h incubation, cell proliferation in each culture condition was evaluated by CCK8 assay. (G)ATP level was measured by an assay kit. Data are presented as mean ± SD from at least three indepen [file 12964_2023_1061_MOESM1_ESM.docx]

**Supplementary Materials**

**This supplementary file includes:**

Materials and Methods

Figs. S1 to S9

Table S1 to S5

References

**Materials and Methods**

**Materials**

We obtained DMEM and FBS from hyclone. Penicillin, streptomycin sulfate, 0.25% Trypsin and OPTI-MEM from Gibco. TRIzol^TM^ Reagent, Lipofectamine 3000^®^ Transfection Reagent from Invitrogen. None-glucose and none-glutamine DMEM,4×Loading buffer, BCA protein assay from Thermo Scientific Pierce. PVDF, ECL from Milipore. PI, SDS, Tris, Tris-Base, glycine, agarose, absolute alcohol, Sanprep Column Plasmid Mini-Preps Kit, Sanprep Column DNA Gel Extraction Kit from Sangon Biotech. 2×Hieff PCR Master Mix, Prime Script™ RT reagent Kit (Perfect Real Time) ,T4 DNA Ligase, DsRed-Express2 fluorescent proteins from takara. XhoI, and NheI were from New England Biolabs. Blood glucose test strips from Ribilda. Tissue DNA kit from Omega. Genomic DNA Buffer Set from Qiagen. SDS-PAGE Gel Kit, Oil Red O solution, Hematoxylin-Eosin Staining Kit, Paraffin with ceresin from Solarbio. 2-NBDG, TNF-α, BSA from Sigma. Lactic Acid assay kit, Glucose Assay Kit, ATP assay kit from Nanjing jiancheng Bioengineering Institute. ELISA Kits from CSB-E05109m, CSB-E05107m, CSB-E05113m, CEA398Ge, CEA458Ge, CEA454Ge, CEA911Ge, E0847M, E0448M.

**Plasmids**

Plasmids used in this study were constructed by standard molecular cloning. The coding region of human CYP17A1 was amplified from HeK293T cell (human emborynic kidney cell 293T line), cDNA using a standard PCR method and cloned into a pcDNA3.0 vector containing a 5×Myc epitope tag. CYP17A1 mutant was generated using Quik Change site-directed mutagenesis (Stratagene). All constructs used in this study were verified by sequencing.

**Cell culture and Transfection**

HeK293T and Huh7 cells purchased from ATCC were grown at a density of 7×10^5^ per 60 mm petri dish at 37 °C in 5% CO_2_. The cells were maintained in medium A (Dulbecco’s modified Eagle’s medium containing 100 units/ml of penicillin and 100 mg/ml of streptomycin sulfate) supplemented with 5% FBS. Medium B (none-glucose or none-glutamine DMEM medium containing 100 units/ml of penicillin and 100 mg/ml of streptomycin sulfate) supplemented with 5% FBS. Twenty-four hour later, all cell lines were transfected with Lipofectamine 3000 reagent according to the manufacturer’s protocol. Cells were harvested 48 hours after transfection.

**Generation of CYP17A1 deletion mutation cell line**

HeK293T cells stably expressing C987X mutation of CYP17A1 gene established by CRISPR/Cas9 technique were generated by EdiGene Inc. (BeiJin). 5’-GGTGAAGAAGAAGCTCTACG-3’ was chosen as targeting guide RNA. Donor (CAGGAGGAGACGGTTACGGTCACTGATAGTTGGTGTGCGGCTGAAACCCACATTCTGGTCAATCTTCTCTAGAGCTTCTTCTTCACCTGAAGACCAGAG)was design and synthesize, then co-transfected with plasmid into 293T cells and seeded into 96-well plates. Clones derived from single cell were validated by sequencing using primer F, 5’-TGGAGACCACCACCTCTGTG-3’; primer R, 5’-CTCCTCTGG GAAGTCAGG A -3’. The pure clone was cultured for subsequent analysis.

**Antibodies**

Primary antibodies used for immunoblots were as follows: rabbit monoclonal anti-Glucose Transporter GLUT1[EPR3915](ab115730), rabbit monoclonal anti-Glucose Transporter GLUT2 [EPR16550](ab192599), rabbit monoclonal anti-Glucose Transporter GLUT3[EPR10508(N)] - N-terminal (ab191071), rabbit monoclonal anti-Glucose Transporter GLUT4 (phospho S488)[EPR930(2)] (ab188317), rabbit monoclonal anti-IGF1 Receptor[EPR19322] (ab182408), rabbit monoclonal anti-HMGCR[EPR1685(N)] (ab174830), rabbit monoclonal anti-LSS[EPR6703] (ab140124), recombinant Rabbit IgG, monoclonal [EPR25A] - Isotype Control (ab172730), goat anti-mouse IgG H&L (HRP) (ab6789), Goat Anti-Rabbit IgG H&L (HRP)(ab6721), mouse monoclonal [9E10] to Myc tag(ab32),rabbit monoclonal [EPR9442(ABC)] to COX IV - Mitochondrial Loading Control(ab202554),mouse monoclonal anti-beta Actin[mAbcam 8226] - Loading Control (ab8226),were all purchased from abcam. Rabbit monoclonal anti-CYP17A1 (E6Y3S) #10443,anti-rabbit IgG Phospho-PI3 Kinase p85 (Tyr458)/p55 (Tyr199) #4228, Phospho-Akt (Ser473) (D9E) XP® rabbit mAb #4060 and GFP (D5.1) XP® Rabbit mAb #2956 were purchased from [Cell Signaling Technology.](https://www.sogou.com/link?url=DSOYnZeCC_qY5nWg7_9-cidQRoztLvNXreaVHN88tHY.)

**Methods**

**Human Subjects**

During the clinical work, a family with inherited coronary heart disease (CAD) were found (Fig.1, A). The affected siblings (II-1, II-2, II-4, III-4, III-8 and III-9) are alive and have received drug treatment. This family is of Chinese Han ancestry, ascertained via subject II-2, who presented with unstable angina and then was admitted to the first affiliated hospital of Xinjiang Medical University. He had smoked and taken alcohol before his first hospitalization. He also had some other risk factors such as type 2 diabetes mellitus and essential hypertension. Both his father and mother had heart disease when they were alive, his mother died of a CAD. All family members with CAD are accompanied by essential hypertension. Detailed clinical data were obtained for all available kindred members, including 6 affected with CAD, 8 free of CAD. Blood biochemical parameters of the family members were measured after fasting overnight. The clinical characteristics of all members are shown in Table S1A-B.

**Clinical validation population**

All participants were recruited in First Affiliated Hospital of Xinjiang Medical University and examined by cardiologists. The coronary artery disease were defined as the presence of at least one significant coronary artery stenosis of ≥50% luminal diameter on coronary angiography. Peripheral blood samples were obtained from CAD patients as well as healthy volunteers. The study was approved by the Ethics Committee of the First Affiliated Hospital of Xinjiang Medical University and informed consent was taken from all the patients. Those with acute and chronic pancreatic/liver/kidney disease, thyroid disease, connective tissue disease, chronic inflammatory disease, and multiple organ failure syndrome as well as those whose data were incomplete were excluded from the study. Participants reporting regular smoking and drinking in the past 6 months were considered as current smokers and alcohol users, respectively. After 10 minutes of rest, blood pressure was measured 3×within 10 minutes, and the mean value was recorded. Essential hypertension was defined as diastolic blood pressure≥90 mmHg or systolic blood pressure≥140mmHg or use of anti-hypertensive medications. Diabetes mellitus was diagnosed based on fasting blood glucose >7.0 mmol/L (126 mg/dL) or 2hour postprandial blood glucose >11.1 mmol/L (200 mg/dL) or receiving anti-diabetic treatments. After 12 hours fasting, plasma biochemical variables, including total cholesterol (TC), triglyceride, glucose, HDL-C (high-density lipoprotein cholesterol), and LDL-C (low-density lipoprotein cholesterol) were measured using a standard method (AR/AVL Clinical Chemistry System). Finally,1028 were included in the study (details see in Table S2).

**Whole-Exome Sequencing and linkage analysis on the early-onset CAD family**

To identify the causal SNP(s), DNA was extracted from peripheral venous blood. Then the samples from subjects with CAD and without CAD were analyzed by whole-exome sequencing (WES). More details about the variant filtration performed from the exome sequencing processing, massively parallel sequencing data processing and SNV/ indel calling, linkage analysis on the Han family had been described previously^[23, 52]^. Schematic of processing in variant identification see in Figure S1.

Whole-Exome Sequencing Genomic sequencing was performed by Shanghai Majorbio Bio-Pharm Technology Co.,Ltd. ( http://www.majorbio.com/). Exomes were captured using the Illumina Truseq exome enrichment kit V3 according to the manufacturer’s manual (Illumina) and sequenced using the Hiseq2000 Sequencing System (Illumina). Sequencing reads were obtained in the Fastq format and aligned to human genome (hg19) using Burrows Wheeler Aligner software. The possible disease-causing variants including single nucleotide polymorphisms and insertions/deletions (indels) were identified using a genome analysis toolkit and VarScan 2.2.7 software. Briefly, heterozygous in case samples and homozygous in control samples were filtered using the autosomal dominant inheritance pattern. Variants with a minor allele frequency <0.5% in Chinese population from 1000 Genomes database, or with a minor allele frequency <0.1% in East Asian populations in ExAC database were selected. Variants were filtered for missense, splice-site, stop-gain or stop-loss and frame shift mutations, and then for deleterious mutations, including those with indels or Combined Annotation Dependent Depletion scores >4. The resulting variant of CYP17A1 gene identified was then validated by sanger sequencing.

**Genetic analysis**

This new mutation of CYP17A1 identified by WES (named C987X) was validated in 576 CAD cases and 452 control subjects. The genomic DNA (100 ng) were used as polymerase chain reaction (PCR) templates. PCR amplification was performed using 10ul of 2×Hieff PCR Master Mix, 1ul of probes, 1ul of DNA template and 8ul ddH2O in a final reaction volume of 20ul. Thermal cycling conditions were as follows: 95°C for 5 min; 36 cycles of 94°C for 30s, 58°C for 30s, 72°C for 40s; and 72°C for 5 min. The SNP were genotyped using direct sequencing method, DNAMan software and Chromas software were used for sequencing results. 10% of the total genotyped samples were duplicated, and there was at least one positive and one negative control per 96-well DNA plate in our study. We obtained 100% concordance between the genotyped duplicate samples for this SNP. The distributions of sequence variants were compared between CAD patients and controls.

**Multiplex PCR and sequencing**

Multiplex PCR and sequencing (Sangon Biotech) were used to identification CAD related polymorphisms in whole exons of CYP17A1. A panel which contains target SNP sites were designed. Library preparation was performed by two step PCR. First round PCR reaction was set up as follows: DNA (10 ng/μl) 2μl; amplicon PCR forward primer mix (10 μM) 1μl; amplicon PCR reverse primer mix (10 μM) 1μl; 2×PCR Ready Mix 15 μl (total 25μl) (Kapa HiFi Ready Mix). The plate was sealed and PCR performed in a thermal instrument (BIO-RAD, T100TM) using the following program: 1 cycle of denaturing at 98 °C for 5 min, first 8 cycles of denaturing at 98 °C for 30 s, annealing at 50 °C for 30 s, elongation at 72 °C for 30 s, then 25 cycles of denaturing at 98 °C for 30 s, annealing at 66 °C for 30 s, elongation at 72 °C for 30 s and a final extension at 72 °C for 5 min. Finally hold at 4℃.The PCR products were checked using electrophoresis in 1 % (w/v) agarose gels in TBE buffer (Tris, boric acid, EDTA) stained with ethidium bromide (EB) and visualized under UV light. Then we used AMPure XP beads to purify the amplicon product. After that, the second round PCR was performed. PCR reaction was set up as follows: DNA (10 ng/μl) 2μl; universal P7 primer with barcode (10 μM) 1μl; universal P5 primer (10 μM) 1μl; 2× PCR Ready Mix 15 μl (total 30μl) (Kapa HiFi Ready Mix). The plate was sealed and PCR performed in a thermal instrument (BIO-RAD, T100TM) using the following program: 1 cycle of denaturing at 98 °C for 3 min, then 5 cycles of denaturing at 94 °C for 30 s, annealing at 55 °C for 20 s, elongation at 72 °C for 30 s, and a final extension at 72 °C for 5 min. Then we used AMPure XP beads to purify the amplicon product. The libraries were then quantified and pooled. Paired-end sequencing of the library was performed on the HiSeq XTen sequencers (Illumina, San Diego, CA).

**Data QC and SNP calling**

Raw reads were filtered according to two steps: 1) Removing adaptor sequence if reads contains by cutadapt (v 1.2.1); 2) Removing low quality bases from reads 3’ to 5’ (Q < 20) by PRINSEQ-lite（v 0.20.3); And the remaining clean data were mapped to the reference genome by BWA (version 0.7.13-r1126) with default parameters. Samtools (Version: 0.1.18) was used to calculate each genotype of target site. Annovar (2018-04-16) was used to detect genetic variants.

**Immunofluorescence**

To label intracellular-localized protein, cells on glass coverslips were fixed with 4% paraformaldehyde for 20 min at room temperature and washed with PBS. Next, the cells were permeabilized in PBS containing 0.2% Triton X-100 for 10 min. Then cells were incubated in blocking buffer (1% BSA in PBS) for 1h at room temperature. Cells were then incubated overnight in blocking buffer containing primary antibodies and additional incubation with fluorescent secondary antibodies in blocking buffer for 2h at room temperature. Cells were analyzed with a Leica confocal scanning laser microscope (Leica SP8) with a 63 oil immersive objective.

**RNA-seq**

RNA isolation

Total RNA was extracted using the Total RNA Extractor（Trizol）kit (B511311, Sangon, China) according to the manufacturer’s protocol, and treated with RNase-free DNase I to remove genomic DNA contamination. RNA integrity was evaluated with a 1.0% agarose gel. Thereafter, the quality and quantity of RNA were assessed using a NanoPhotometer ® spectrophotometer (IMPLEN, CA, USA) and an Agilent 2100 Bioanalyzer (Agilent Technologies, CA, USA). The high quality RNA samples were subsequently submitted to the Sangon Biotech (Shanghai) Co., Ltd. for library preparation and sequencing.

Library preparation and sequencing

A total amount of 2 μg RNA per sample was used as input material for the RNA sample preparations. Sequencing libraries were generated using VAHTSTM mRNA-seq V2 Library Prep Kit for Illumina® following manufacturer’s recommendations and index codes were added to attribute sequences to each sample. Briefly, mRNA was purified from total RNA using poly-T oligo-attached magnetic beads. Fragmentation was carried out using divalent cations under elevated temperature in VAHTSTM First Strand Synthesis Reaction Buffer (5X). First strand cDNA was synthesized using random hexamer primer and M-MuLV Reverse Transcriptase (RNase H-). Sond strand cDNA synthesis was subsequently performed using DNA polymerase I and RNase H. Remaining overhangs were converted into blunt ends via exonuclease/polymerase activities. After adenylation of 3’ ends of DNA fragments, adaptor was ligated to prepare for library. In order to select cDNA fragments of preferentially 150~200 bp in length, the library fragments were purified with AMPure XP system (Beckman Coulter, Beverly, USA). Then 3 μL USER Enzyme (NEB, USA) was used with size-selected, adaptor-ligated cDNA at 37°C for 15 min followed by 5 min at 95 °C before PCR. Then PCR was performed with Phusion High-Fidelity DNA polymerase, Universal PCR primers and Index (X) Primer. At last, PCR products were purified (AMPure XP system) and library quality was assessed on the Agilent Bioanalyzer 2100 system. The libraries were then quantified and pooled. Paired-end sequencing of the library was performed on the HiSeq XTen sequencers (Illumina, San Diego, CA).

Data assessment and quality control

FastQC (version 0.11.2) was used for evaluating the quality of sequenced data. Raw reads were filtered by Trimmomatic (version 0.36) according to several steps: 1) Removing adaptor sequence if reads contains; 2) Removing low quality bases from reads 3’ to 5’ (Q < 20); 3) Removing low quality bases from reads 5’ to 3’ (Q < 20); 4) Using a sliding window method to remove the base value less than 20 of reads tail (window size is 5 bp); 5) Removing reads with reads length less than 35 nt and its pairing reads. And the remaining clean data was used for further analysis.

Alignment with reference genome

Clean reads were mapped to the reference genome by HISAT2 (version 2.0) with default parameters. RSeQC (version 2.6.1) was used to statistics the alignment results. The homogeneity distribution and the genome structure were checked by Qualimap (version 2.2.1). BEDTools (version 2.26.0) was used to statistical analysis the gene coverage ratio.

Expression analysis

Gene expression values of the transcripts were computed by StringTie (version 1.3.3b). Principal Component Analysis (PCA) and Principal co-ordinates analysis (PCoA) were performed to reflect the distance and difference between samples. The TPM (Transcripts Per Million), eliminates the influence of gene lengths and sequencing discrepancies to enable direct comparison of gene expression between samples. DESeq2 (version 1.12.4) was used to determine differentially expressed genes (DEGs) between two samples. Genes were considered as significant differentially expressed if q-value <0.001 and |Fold Change| >2. When the normalized expression of a gene was zero between two samples, its expression value was adjusted to 0.01 (as 0 cannot be plotted on a log plot). If the normalized expression of a certain gene in two libraries was all lower than 1, further differential expression analysis was conducted without this gene. Gene expression differences were visualized by scatter plot, MA plot and volcano plot.

Functional analysis of differentially expressed genes

Functional enrichment analyses including Gene Ontology (GO) and KEGG was performed to identify which DEGs were significantly enriched in GO terms or metabolic pathways. Gene Ontology (GO) is an international standard classification system for gene function. DEGs are mapped to the GO terms (biological functions) in the database, the number of genes in every term is calculated, and a hypergeometric test is performed to identify significantly enriched GO terms in the gene list out of the background of the reference gene list. The Kyoto Encyclopedia of Genes and Genomes (KEGG) database is a public database of pathway data, KEGG pathway analysis identifies significantly enriched metabolic pathways or signal transduction pathways enriched in DEGs compared to a reference gene background, using the hypergeometric test. GO terms and KEGG pathway with false discovery rate (q-value) < 0.05 were considered as significantly altered.

**Cell proliferation assay by CCK8**

CCK-8 assay was used to evaluate cells proliferation. Briefly, HEK293T cells with transient CYP17A1 WT and C987X over-expressed were plated onto 96-well plates. At 24h post seeding, 10μl CCK-8 reagent was added to the wells and incubated for 1h. The absorbance was determined at 450 nm using a SpectraMax 190 Microplate Reader (Molecular Devices).

**Glucose uptake measurement by flow cytometry analysis of 2-NBDG**

Add 10 ng/mL TNF-α to the transient transfection of 293T cells, after 48h preincubation, all culture medium was removed from each well and replaced by culture medium with 100 μM 2-NBDG.45mins later, the 2-NBDG uptake reaction was stopped by removing the incubation medium and washing the cells twice with pre-cold phosphate buffered saline (PBS). Cells in each well were subsequently resuspended in pre-cold fresh growth medium and then given a final Propidium Iodide (PI) concentration of 1μl/ml and maintained at 4℃ for later flow cytometry analysis performed within 30 min. For each measurement, data from 2000 single cell events was collected using a FACScalibur (Becton Dickinson Immunocytometry Systems, SanJose, CA) flow cytometer within 20 s.

**Co-Immunoprecipitation**

Cells were harvested and lysed in 600μL of lysis buffer (150 mmol/L NaCl, 1 mmol/L EDTA, 0.5% Nonidet P-40) supplemented with protease inhibitors. Lysates were cleared by centrifugation at 13200g for 10 minutes at 4℃. Supernatants were then incubated with 40 μL of the anti-CYP17A1 affinity gel for 4 hours at 4℃. Beads were washed 5×with ice-cold lysis buffer. Bound proteins were eluted by boiling in 2× loading buffer at 95℃ for 10 minutes and analyzed by immunoblotting.

**Mass spectrometry**

Mass spectrometry procedures were performed as previously described ^[52]^. The LC-MS/MS analysis was performed by LIFEINT TECHNOLOGY CO., LTD.

**Ubiquitination Assays**

Cells were harvested and lysed in IP buffer (1×PBS containing 1% Nonidet P-40,5mmol/L EDTA, 5mmol/L EGTA, 0.1 mmol/L leupeptin, 1% (w/v) deoxycholate) supplemented with protease inhibitors plus 10 mmol/L N-ethylmaleimide and 10μmol/L MG132. Lysates were cleared by centrifugation, and supernatants were incubated with the anti-N-Myc affinity gel for 4 hours at 4°C. Beads were washed 3×with ice-cold IP buffer and boiled at 95°C for 10 minutes in the DTT-free loading buffer. Eluates were analyzed by immunoblotting.

**RNA Isolation and Quantitative Polymerase Chain Reaction**

Total RNA was isolated from cells using Trizol. Reverse transcription was primed with Oligo dT. Quantitative real-time polymerase chain reaction using target specific primers was performed in the Stratagene Mx3000 quantitative real-time polymerase chain reaction Systems. Each sample was done in triplicate, and the relative amounts of mRNAs were calculated using the double delta CT method.

**Immunoblot analysis**

For whole cell lysate, the cells were harvested and suspended with 120 μl of RIPA buffer supplemented with protease inhibitors and then passed 10 times through a number 7 needle. Protein concentration of the extracts was determined according to the BCA method, and then the extracts were mixed with SDS loading buffer. After boiling for 10 min, the extracts were subjected to SDS-PAGE, transferred to nitrocellulose filters. Membranes were blocked by TBS–Tween (1‰) supplemented with 5% skim milk for 1 h at room temperature and then incubated with the indicated primary antibodies overnight at 4 °C. Membranes were washed three times with TBS–Tween and incubated with secondary antibodies (with proportional dilution) diluted in TBS–Tween supplemented with 5% skim milk for 1 h at room temperature, followed by at least three washes with TBS–Tween, finally subjected to immunoblot analysis. Quantification of the immunoblot was performed with ImageJ.

**Animals and treatments**

The heterozygous of CYP17A1 whole-body knockout mice were generated by Cyagen Biosciences Inc. (Guangzhou, China) using customized TALENs. [ApoE-knockout](E:/Program%20Files%20(x86)/Youdao/Dict/8.8.1.0/resultui/html/index.html#/javascript:;) (ApoE^-/-^) mice were purchased from Shanghai Biomodel Organism Science & Technology Development Co.,Ltd. As the CYP17A1 homozygous cann’t fertile, the CYP17A1 heterozygous mice (CYP17A1^+/-^) and ApoE^-/-^ homozygous mice(F1) were crossbred to generate CYP17A1^+/-^ApoE^+/-^ (F2). The F2 progeny were crossbred for generating objective mice. The wild-type (WT) background of each of the lines was C57/BL6. Homozygous offspring as well as their WT siblings were used for the following experiments. Thereby the studied animals identical in their genetic and epigenetic background with the exception of the knockout of the CYP17A1 or/and ApoE gene. Animals were maintained under controlled conditions of temperature (20±1◦C, relative humidity 50–60%), illumination (12h light, 12h dark). Mice were allowed ad libitum access to water and a standard laboratory diet (Beijing HFK Bioscience number 1026, protein ≥18%, fat ≥4%, fiber ≤5%, ash ≤8%, moisture ≤10%, lysine ≥0.82%, calcium =1.0%–1.8%, phosphorus =0.6%–1.2%, and salt =0.3%–0.8%) or a customized feeding diet containing 84.75% basic maintenance feed, 15% lard, and 0.25% cholesterol (Jiangsu synergetic pharmaceutical biological engineering co. LTD). All experiments were performed in accordance with the Provisions and General Recommendations of the Chinese Experimental Animal Administration Legislation, as well as institutional approval from the Xinjiang Medical University Experimental Animal Ethics Committee.

**Generation and genotyping of mice**

All offspring were genotyped by PCR amplification. Genomic DNA were extracted from 2-5mm of tail tissue collected under anesthesia (halothane) from 4-wk old mice by using DNA extraction kit (Omega). To determine ApoE genotype, the common forward primer (GCCTAGCCGAGGGAGAGCCG) was used, reverse primer (TGTGACTTGGG AGCTCTGCAGC) to amplify a 155-bp product in the WT allele or reverse primer (GCCGCCCCGACTGCATCT) to amplify a 245-bp product in the knockout allele. To determine CYP17A1 genotype, forward primer (TGCTGAAATTGCTGTAGCTTCTCCA) and reverse primer (TTGAGGGAGCA GTCCCACAAGTC) were used by direct sequencing. Mice were followed for 3, 6, 9 and 12 months. Each genotype and age group consisted up to 5-8 mice.

**Glucose and insulin tolerance tests**

Glucose tolerance tests were performed after a 14h fast, with 2g/kg glucose administered via oral gavage (n=5 mice per group). Peripheral plasma glucose levels were determined from blood samples taken from the tail vein using an automated glucometer (eB-G, Ribilda, Taiwan) prior to oral glucose administration and 20, 40, 60, 90, and 120 min post-glucose gavage. Intraperitoneal insulin tolerance tests were performed on random-fed mice injected with insulin (0.75 U/kg body weight, Actrapid; Novo Nordisk, Auckland, New Zealand), where peripheral plasma glucose levels from blood samples taken from the tail vein were measured before and 20, 40, 60, 90, and 120 min after insulin injection (n=5 mice per group). The glucose level at the 0-min time point was presented as 100%, and glucose levels recorded at the subsequent time points were calculated as the percentage of the 0-min time point.

**Blood biochemistry**

Blood was collected by cardiac puncture from animals after euthanasia, and plasma (n =6 mice/ group) samples were stored at -80°C until analyzed. Plasma AST and ALT levels were measured by fast automatic biochemical analyzer .DHEA , DHEAS, plasma [Estrogen](E:/Program%20Files%20(x86)/Youdao/Dict/8.9.3.0/resultui/html/index.html#/javascript:;), plasma aldosterone, plasma cortisol, plasma insulin，plasma resistin, and plasma 17-hydroxyprogesterone were measured by ELISA kits on a Luminex200 (Austin, TX) according to the manufacturer’s instructions.

**Tissue collection, hepatic and gonadal adipose histology**

Following euthanasia, whole liver and gonadal fat pads were separated from connective tissue and wet weights recorded. Tissues were treated differently according to experimental requirements. The left lobe of the liver and regions of the gonadal fat pads were routinely fixed in 10% phosphate buffered formalin (pH 7.4), embedded in paraffin and stored in 70% alcohol at 4℃, other tissue sections were snap frozen in liquid nitrogen and stored at -80℃ refrigerator. Histological analysis was performed on paraffin sections (Leica Microsystems), stained with hematoxylin and eosin (H&E) and photographed with a Leica digital camera (DFC420).

**Quantitative analysis of atherosclerotic area**

For the atherosclerosis study, whole hearts, with the aortic arch and thoracic aorta attached, were taken and fixed in 10% neutral buffered formalin for at least 24 h. The ventricles were trimmed from the atria and aorta in a plane in line with the tips of the atria. The upper region of the heart was then transferred to formalin containing 10% sucrose overnight at 4°C before being embedded in OCT medium (Miles; Elkhart, IN) and frozen at -80°C. To assess aortic lesion location and size, the aortas of all mice were sectioned, and every fifth 8-_x0005_m section starting from the aortic valve throughout the aortic sinus and down the thoracic branch was collected. Sections were stained using Oil red O, counterstained with Harris’ cold hematoxylin, and examined under bright-field illumination (Olympus BX50). The atherosclerotic lesion area was quantified using NIH Image software (Image J 1.4; http://rsb.info.nih.gov/ij) and presented as percentage of total vessel area. Dissection of aortic tissue under a microscope, and the changes of atherosclerosis. plaque size were observed by oil red O staining.

**Statistical Analysis**

Clinical data were collected and verified by 2 staff members using EpiData3.02 software (EpiData Association). Statistical analyses were performed using GraphPad 6.0 and SPSS version 22.0 software. All data were assessed for normality (Kolmogorov-Smirnov test) and equal variance tests. Continuous variables are expressed as mean±SD in case of normal distribution and as the median (interquartile range) in case of non-normal distribution. Continuous variables were analyzed by Student t test or one-way ANOVA with the indicated post hoc test for grouped analyses. Non-normally distributed variable were analyzed by Mann–Whitney U test as indicated. Categorical variables are reported as counts (percentages), and the differences were analyzed by χ^2^ test. Statistical significance was set at P<0.05. Sample sizes, statistical tests, and P values for each experiment are depicted in the relevant tables and figure legends.

**
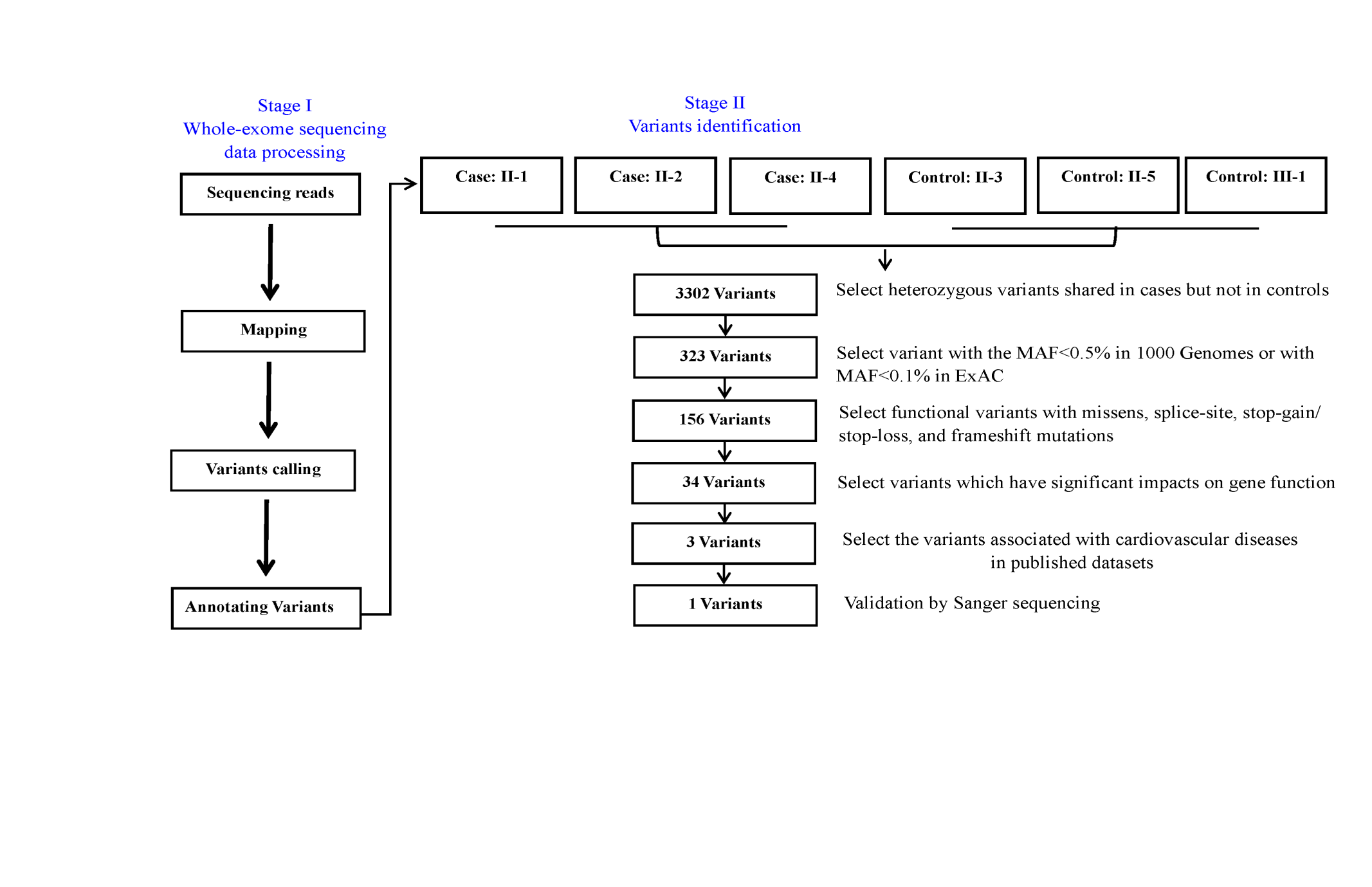
**

**Figure S1.** Schematic of whole-exome sequencing data processing and variant identification.

Fig.S1. Schematic of whole-exome sequencing data processing and variant identification.

A


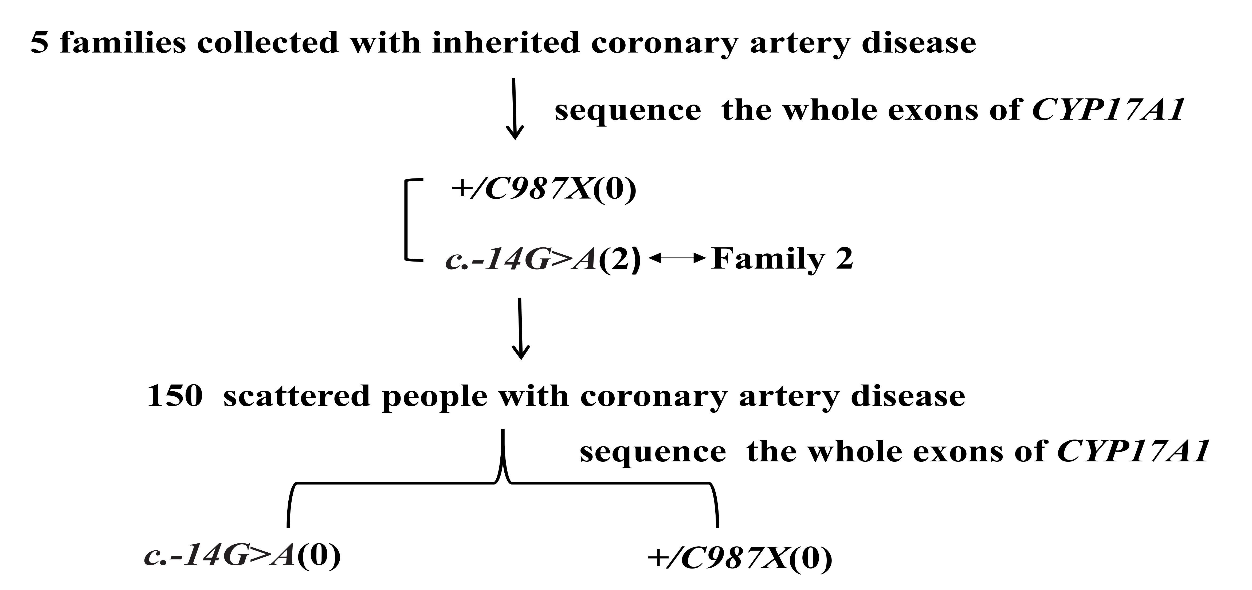


B


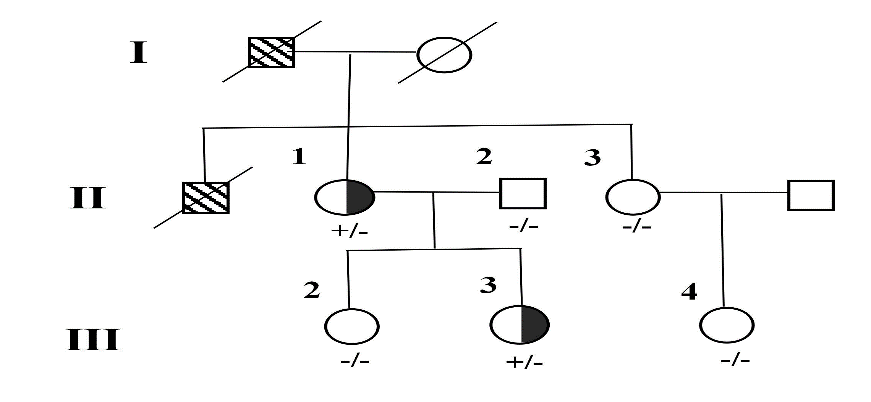


**Figure. S2.** The c.-14G>A variant in *CYP17A1*.

**(A)**Schematic of identification CYP17A1 as a candidate gene for CAD susceptibility.

**(B)** Pedigrees of the family 2 with c.-14G>A mutation. CYP17A1 genotype (half filled for c.-14G>A carriers, open for wild-type individuals) are shown below each square or circle.





D

C

B

A


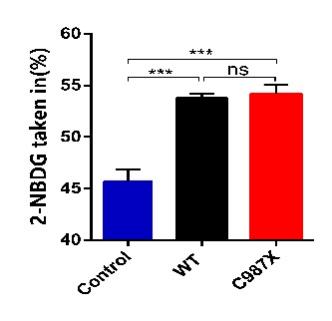

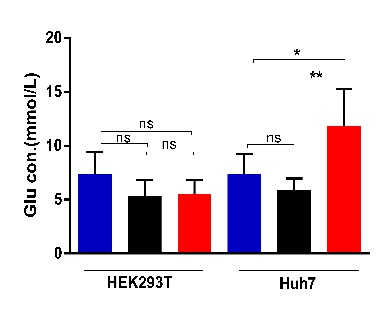

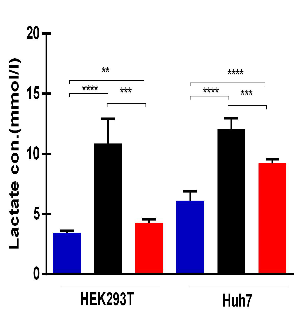

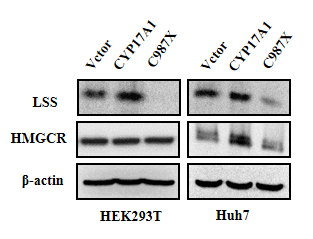

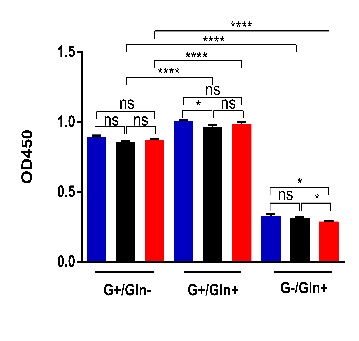

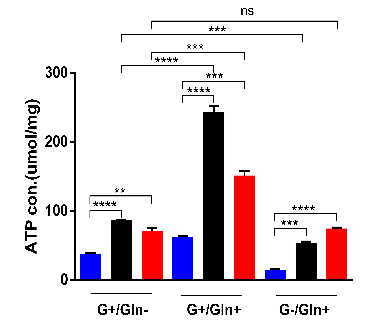


H

F

E

G


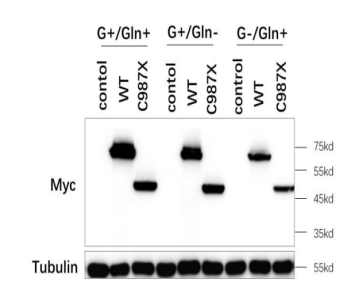


**Figure** S3. CYP17A1 over-expression involved in glycometabolism.

**(A)**Relative mRNA levels of genes related to glycolytic pathway (screened by RNA-seq) in stably expressing C987X mutation or in HEK293T cells with over-expressed CYP17A1 WT or C987X.Vector as control.

**(B)**CYP17A1 promotes the glucose uptake in HEK293T cells. HEK293T cells transfected with plasmids were labeled with intracellular level of 2-NBDG and subjected to measurement of flow cytometry.

HEK293T cells cultured in a 6-well culture plate were transiently transfected with 3μg/well of plasmids encoding CYP17A1(WT) or CYP17A1(C987X). After 48h transfection, supernatants were harvested and subjected to the detection of glucose**(C)** or lactate**(D)** using assay kits.

**(E)**After 48h transfection, cell lysates were prepared and subjected to western blot analysis. β-actin expression was used as a loading control. The effect of CYP17A1 and C987X mutation on key enzymes of cholesterol synthase in HEK293T and Huh7 cells were detected.

**(F)**The influence of glucose and glutamine on CYP17A1-mediated cell proliferation. HEK293T cells with transient CYP17A1 WT or C987X expression were cultured in three different media: the complete medium with both glucose (Glu+) and glutamine (Gln+); the culture medium with glucose (Glu+) but without glutamine (Gln-); the culture medium without glucose (Glu-) but with glutamine (Gln+). After 24h incubation, cell proliferation in each culture condition was evaluated by CCK8 assay.

**(G)**ATP level was measured by an assay kit. Data are presented as mean ± SD from at least three independent experiments.

**(H)**The expression of CYP17A1 protein in different media, detection by WB. n=3 biological repetition. *, p < 0.0.5; **,p < 0.01; ***, p < 0.005，****, p < 0.001.n.s., no significance.

**HEK293T cell transfected with plasmids**

**Harvested 48h later**

**WT sample IP**

**with anti-CYP17A1**

**C987X sample IP with anti-CYP17A1**

**WT sample IP**

**with anti-IgG**

**Washing**

**Elute**

**MS/MS**

**Proteins identified in WT**

| **No.** | **Accession** | **Protein name** |
| --- | --- | --- |
| **1** | **P35908** | **Keratin, type II cytoskeletal 2 epidermal** |
| **2** | **P04264** | **Keratin,type II cytoskeletal 1** |
| **3** | **P20700** | **Lamin-B1** |
| **4** | **P25705** | **ATP synthase subunit alpha** |
| **5** | **P07437** | **Tubulin beta chain** |
| **6** | **P35527** | **Keratin,type I cytoskeletal 9** |
| **7** | **O15381** | **Nuclear valosin-containing protein-like** |
| **8** | **P68371** | **Tubulin beta-4B chain** |
| **9** | **P13645** | **Keratin,type I cytoskeletal 10** |
| **10** | **P09874** | **Poly [ADP-ribose] polymerase 1** |
| **11** | **Q08211** | ***ATP-dependent RNA helicase A*** |
| **12** | **P08238** | **Heat shock protein HSP 90-beta** |
| **13** | **P34896** | **Serine hydroxymethyltransferase** |
| **14** | **P11142** | **Heat shock cognate 71 kDa protein** |
| **15** | **Q13885** | **Tubulin beta-2A chain** |
| **16** | **P06576** | **ATP synthase subunit beta** |
| **17** | **P0DMV9** | **Heat shock 70 kDa protein 1B** |
| **18** | **P08670** | **Vimentin** |
| **19** | **P42704** | **Leucine-rich PPR motif-containing protein** |
| **20** | **P14618** | **Pyruvate kinase PKM** |

**Figure S4**. Identification of CYP17A1-binding proteins. **(A)**Schematic of the work flow of immunoprecipitation (CO-IP) followed by tandem mass spectrometry (MS/MS). **(B)** The top twenty candidate proteins that specifically identified in anti-CYP17A1 IP from HEK293T cell transfected with WT plasmids sample shown in (A).

**HEK293T cell transfected with plasmids**

**Harvested 48h later**

**Washing**

**Elute**

**MS/MS**

**WT plasmid**

**C987X plasmid**

**A**

**Alignment with reference genome**

**Proteins identification**

**HeK293T cells stably expressing C987X mutation**

**WT sample*3**

**C987X sample *3**

**RNA isolation**

##### Library preparation and sequencing

##### Data assessment and quality control

**Alignment with reference genome**

**CO-IP**

**RNA-seq**

**Combine analysis**

**Differential expression genes**

**Determined in mRNA expression**

**B**

| **Parts of functional paths** | **Differences in genes involved** |
| --- | --- |
| **Glycosyl compound metabolic process** | **ADK\|AK4\|AKR1B1\|GLA\|PRPS1** |
| **Metabolism of steroids** | **AKR1B1\|IDI1\|OSBPL6** |
| **Regeneration** | **ANXA1\|FOLR1\|GAS6\|GSTP1\|LCP1\|TYMS\|PNPT1** |
| **Ribosome biogenesis** | **DKC1\|RRP1\|RRP9\|UTP14A\|RRP15\|TFB1M\|NOP16** |
| **Pyruvate metabolic process** | **DLAT\|HK2\|PCK2\|NUP205\|NUP35** |
| **Glycolysis / Gluconeogenesis** | **DLAT\|HK2\|PCK2** |
| **Glycerolipid biosynthetic process** | **PCK2\|PIGK\|TAMM41** |

**C**


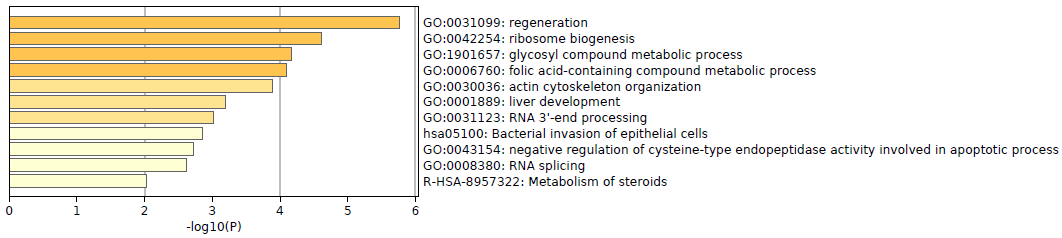


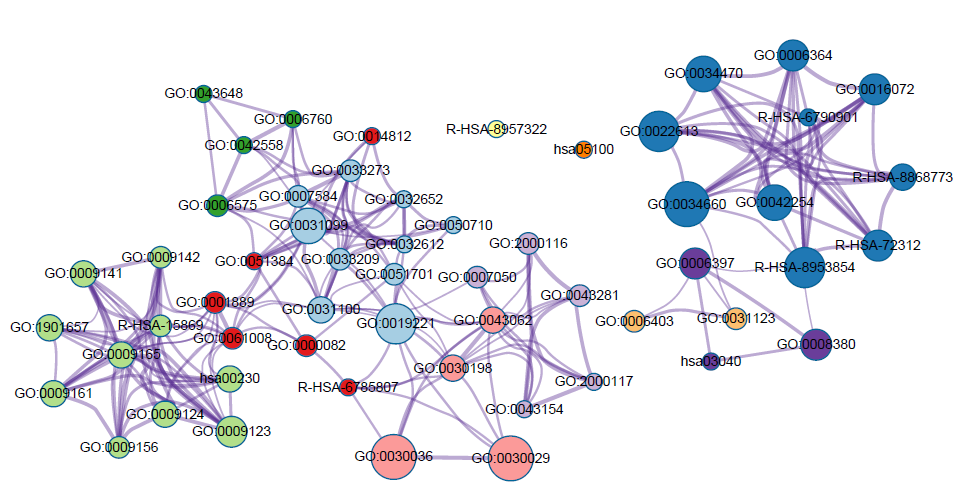

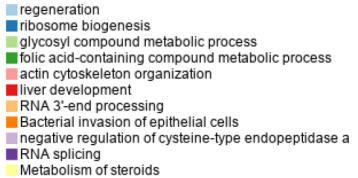


**D**

**E**


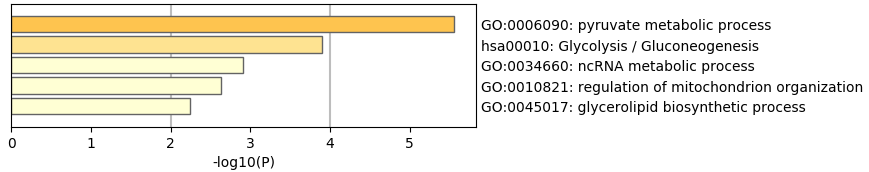


**F**


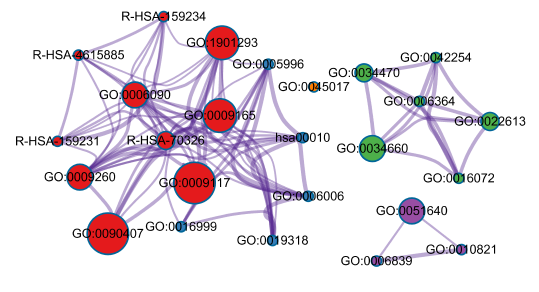

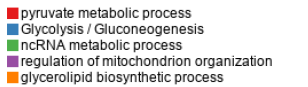


**G**


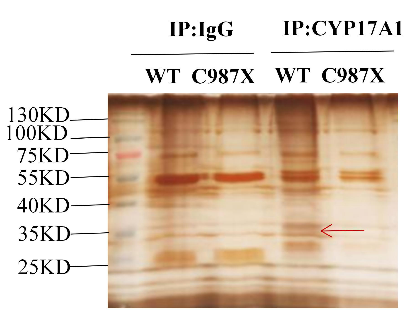


**Figure S5.** Identification of CYP17A1 functional paths.

**(A)**Schematic of the work flow of combine analysis of CO-IP and RNA-seq. **(B)** Parts of candidate gene that specifically related to functional paths shown in (A). **(C-F)** Functional path clusters (the color represents the difference, the deeper the more significant) and interaction network (the color of different nodes represents different clusters, the connection line represents the genetic similarity between terms, and the size of nodes represents the number of genes enriched) in differences unique to wild-type (C and D) or to C987X truncation mutant (E and F). **(G)**Silver staining of IP. Specific protein (about 35 kD) between CYP17A1 and CYP17A1∆ECD were pointed with an arrow.


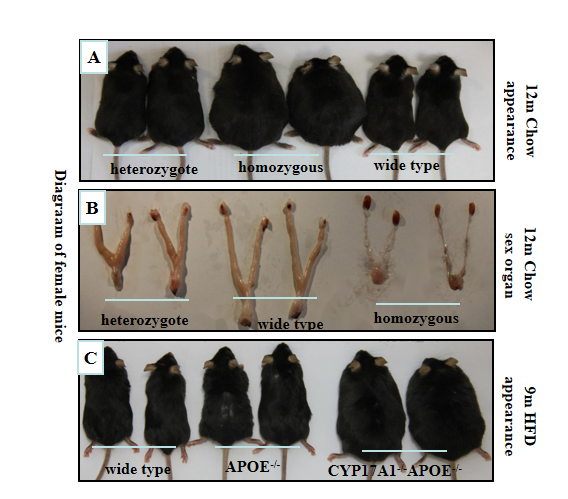

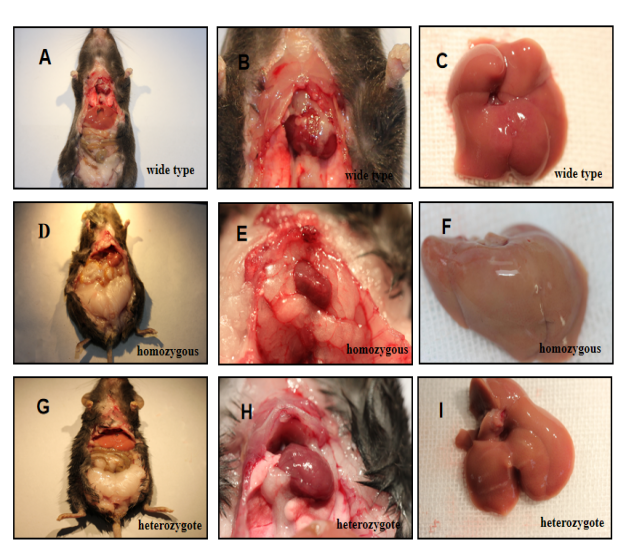


**Figure S6**. Diagram of CYP17A1^-/-^mice. **Upper part: (A)** Representative photos of of appearance(A) in chow fed CYP17A1^-/-^ heterozygote, homozygous and WT mice at 12 mo of age and relative diagram of the anatomy (B), 9 month of HFD appearance(C) of WT, CYP17A1^-/-^ and CYP17A1^-/-^ApoE^-/-^ mice.

**Lower part:** Abdominal, epicardial and liver necropsy photos of representative 12-mo-old CYP17A1^-/-^ heterozygote, homozygous and WT mice, showing increased adiposity in CYP17A1^-/-^ vs. WT mice. Livers of CYP17A1^-/-^ mice tend to display increased fat accumulation displaying yellow coloration compared with WT (F vs. C).

B

A


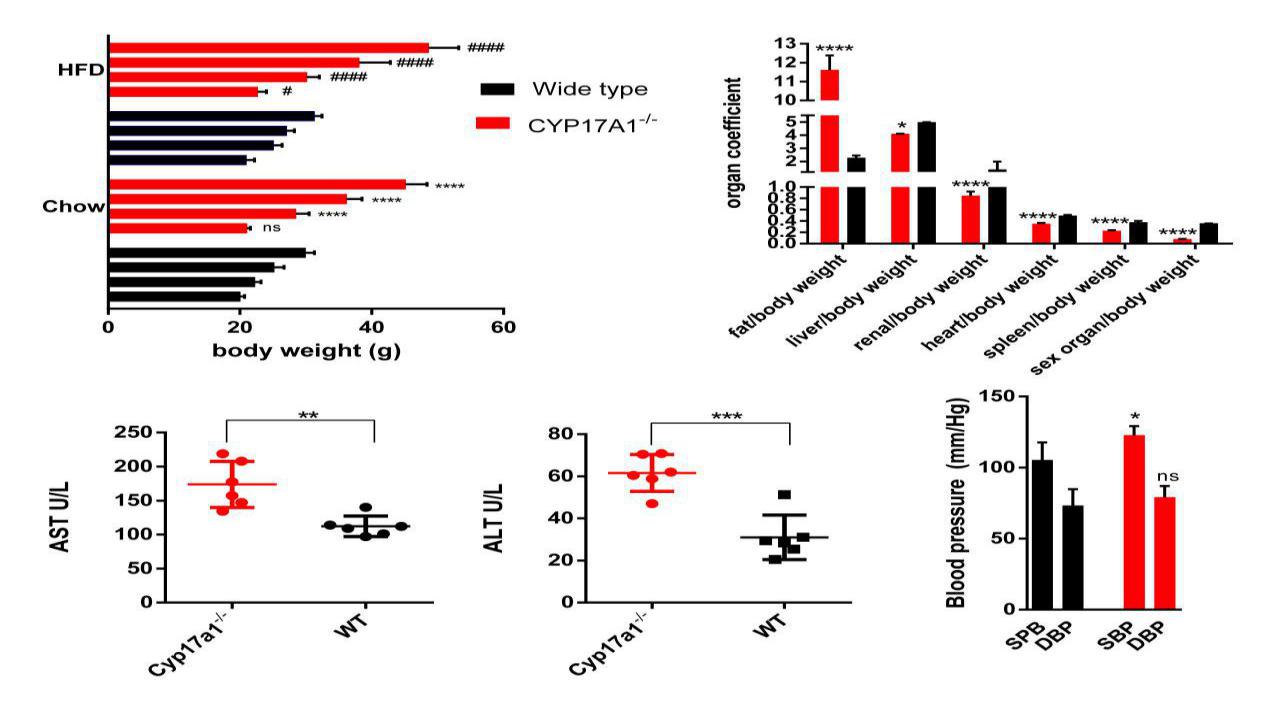


CD

D

E

**Figure S7.** Characterization of CYP17A1 whole-body knockout mice. **(A)**WT, CYP17A1^-/-^ mice (n=8) were fed on chow diet or HCD (0.25% cholesterol). Weights were measured at 3,6,9,12 month. Data is expressed as mean ± SD. Statistical analyses, two-way ANOVA. **(B)**Tissues taken from 12-mo-old WT and CYP17A1^-/-^ mice were immediately weighted by micro balance. Organ coefficient was calculated.

Biochemical detector to detect serum AST**(C)** and ALT**(D)**. **(E)**Blood pressures were measured indirectly by tail arteries in awake mice. Data are expressed as median with SD. Nonparametric test. ^#^P<0.05, ^##^P<0.01, ^###^P<0.001, ^####^P<0.0001, compared with WT mice fed with HCD diet. *P<0.05, **P<0.01, ***P<0.001, ****P<0.0001, compared with WT mice fed with chow diet. ns, not statistically significant compared with WT mice fed with chow diet. NS, not statistically significant compared with WT mice fed with HCD.


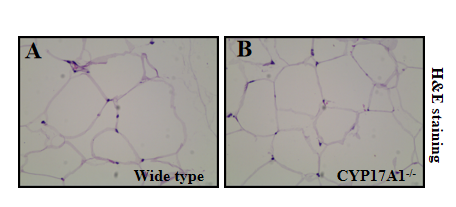

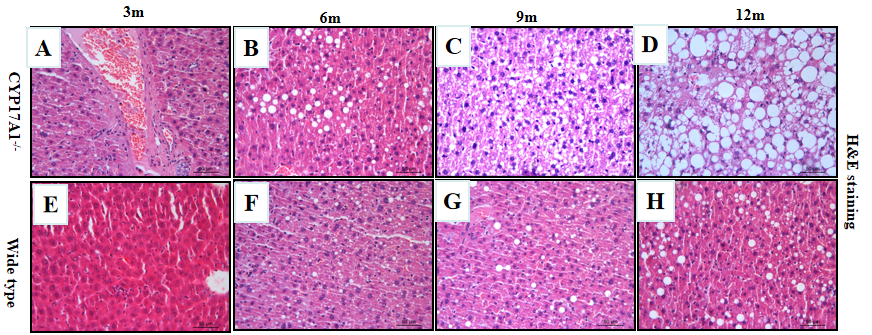


**Figure S8.** Adipose and liver histology in CYP17A1^-/-^ mice. Upper part: Hematoxylin and eosin (H&E) staining of gonadal adipose sections from WT (A), CYP17A1^-/-^(B) mice at 12 mo of age. Lower part: H&E staining of liver sections. H&E staining of liver sections from chow fed female CYP17A1^-/-^ mice at 3, 6,9 and 12 mo-old (A-D). WT female mice at 3, 6, 9and 12 mo(E-H), respectively. All histology images are X40 magniﬁcation; scale bar in bottom right =50μm.

**Figure S9.** Metabolic characteristics of CYP17A1 whole-body knockout mice. WT and CYP17A1^-/-^ mice at 6 or 12mo-old (n=6, each month/each genotype) with chow diet, WT and CYP17A1^-/-^ mice a t6 or 9 mo-old (n=6, each month/each genotype with HCD. Plasma DHEA, DHEAS, plasma [Estrogen](E:/Program%20Files%20(x86)/Youdao/Dict/8.9.3.0/resultui/html/index.html#/javascript:;),plasma aldosterone, plasma cortisol, plasma insulin, plasma resistin and plasma 17-hydroxyprogesterone were measured. Data are expressed as mean ± SD. Statistical analyses, two-way ANOVA. ^#^P<0.05, ^##^P<0.01, ^###^P<0.001, ^####^P<0.0001, compared with WT mice fed with HCD diet. *P<0.05, **P<0.01, ***P<0.001, ****P<0.0001, compared with WT mice fed with chow diet.

**Table S1A. Information of the members of a Chinese Han family with inherited CAD.**

**Table S1B. The traits of heterozygote and wide type of the family members.**

| **Trait** | **Heterozygote (n=6)** | **Wide type (n=8)** | ***P* value** |
| --- | --- | --- | --- |
| Urea nitrogen | 5.00±1.57 | 5.20±0.99 | 0.79 |
| Creatinine | 75.83±10.83 | 78.75±16.35 | 0.70 |
| Uric acid | 305.83±68.90 | 322.88±40.84 | 0.61 |
| TG | 1.58±0.40 | 3.22±1.08 | 0.003 |
| TC | 3.62±0.94 | 3.70±1.29 | 0.91 |
| HDL-C | 0.86±0.34 | 1.11±0.68 | 0.37 |
| LDL-C | 2.33±0.75 | 1.85±0.66 | 1.25 |

TG: triglyceride; TC: total cholesterol; HDL-C: high-density lipoprotein cholesterol; LDL-C: low-density lipoprotein. Comparison of phenotypes in carriers and noncarriers of CYP17A1 C987X. Means ± standard deviation was shown for quantitative traits.

**Table S2. The baseline characteristics of clinical validation population**

| Characteristics | Control (n=452) | CHD (n=576) | χ2 or t | *P* value |
| --- | --- | --- | --- | --- |
| Age, mean (SD) | 54.00(11.70) | 59.00(11.12) | 6.989 | <0.001 |
| Male, n (%) | 229(50.7) | 427(74.1) | 60.485 | <0.001 |
| Smoking, n (%) | 122(33.3) | 289(65.8) | 84.357 | <0.001 |
| Drinking, n (%) | 112(24.8) | 201(34.9) | 12.241 | <0.001 |
| Family history, n (%) | 8(1.8) | 20(3.5) | 2.77 | 0.096 |
| Hypertension, n (%) | 166(36.7) | 323(56.1) | 38.023 | <0.001 |
| Diabetes, n (%) | 49(10.8) | 162(28.1) | 46.382 | <0.001 |
| TG, mean (SD) | 1.77 (1.36) | 1.99(1.67) | 2.165 | 0.031 |
| TC, mean (SD) | 4.05 (1.07) | 3.70(1.19) | 4.835 | <0.001 |
| HDL-C, mean (SD) | 1.16 (0.36) | 1.06(0.71) | 2.634 | 0.009 |
| LDL-C, mean (SD) | 2.61 (0.83) | 2.45(3.26) | 0.986 | 0.324 |
| APOA-I, mean (SD) | 1.21(0.27) | 1.08 (0.28) | 7.511 | <0.001 |
| Lp-A, mean (SD) | 174.34 (159.56) | 222.60(206.63) | 3.948 | <0.001 |

**Table S3. The lists of sgRNA**.

| NO. | Primers |
| --- | --- |
| SgRNA 1 | GGTGAAGAAGAAGCTCTACG |
| SgRNA 2 | CGAGGAGATTGACCAGAATG |
| SgRNA 3 | AGGTGAAGAAGAAGCTCTAC |
| SgRNA 4 | TGAAGAAGAAGCTCTACGAG |
| SgRNA 5 | AGCTCTACGAGGAGATTGAC |
| SgRNA 6 | TTCTGGTCAATCTCCTCGTA |
| SgRNA 7 | CATTCTGGTCAATCTCCTCG |

**Table S4. The primers of glycolytic pathway genes**.

| Gene | Primers |
| --- | --- |
| PGD  F | GGTGCACAACGGGATAGAGT |
| PGD  R | CCATCGGTGTCTTGGAACTT |
| TKT  F | GGAACTAGCCGCCAATACAA |
| TKT R | ACGCGGATGTTGATCTTTTC |
| GPI  F | CGCCCAACCAACTCTATTGT |
| GPI  R | GGTAGAAGCGTCGTGAGAGG |
| G6PD  F | GAGGCCGTGTACACCAAGAT |
| G6PD R | AGCAGTGGGGTGAAAATACG |
| PFP  F | CCTGCACCCTCTGTGAAAAT |
| PFP  R | GGCTTAGGAGTCACGTCCAG |
| RBKS  F | TGGATCCCCAGTTCTACACC |
| RBKS  R | GCTTTGGCTCAGGTTCTGTC |

**Table S5. The differential protein identification by tandem mass spectrometry**.

| **Accession** | **Protein name** | **Fold change** | **Accession** | **Protein name** | **Fold change** |
| --- | --- | --- | --- | --- | --- |
| Q07866 | KLC1 | 15.00 | O00232 | PSMD12 | 0.18 |
| Q9NRA8 | EIF4ENIF1 | 15.00 | Q9Y262 | EIF3L | 0.18 |
| P15924 | DSP | 12.50 | P08240 | SRPRA | 0.17 |
| Q9BRS2 | RIOK1 | 10.50 | P27694 | RPA1 | 0.17 |
| P46013 | MKI67 | 10.00 | P46977 | STT3A | 0.17 |
| P29966 | MARCKS | 9.00 | P82650 | MRPS22 | 0.17 |
| P00492 | HPRT1 | 8.00 | Q5JPE7 | NOMO2 | 0.17 |
| P28838 | LAP3 | 8.00 | Q8TEX9 | IPO4 | 0.17 |
| Q13242 | SRSF9 | 8.00 | Q92900 | UPF1 | 0.17 |
| P12081 | HARS1 | 7.00 | Q9H857 | NT5DC2 | 0.17 |
| P15531 | NME1 | 7.00 | Q9NRG9 | AAAS | 0.17 |
| Q9Y266 | NUDC | 7.00 | Q9NZ01 | TECR | 0.17 |
| P28066 | PSMA5 | 6.00 | Q9Y512 | SAMM50 | 0.17 |
| P31944 | CASP14 | 6.00 | Q9Y512 | SAMM50 | 0.17 |
| P49591 | SARS1 | 6.00 | Q9P2J5 | LARS1 | 0.15 |
| P60174 | TPI1 | 6.00 | O95573 | ACSL3 | 0.14 |
| P60900 | PSMA6 | 6.00 | P26640 | VARS | 0.14 |
| P63241 | EIF5A | 6.00 | Q13620 | CUL4B | 0.14 |
| Q00688 | FKBP3 | 6.00 | Q9H9A6 | LRRC40 | 0.14 |
| Q16629 | SRSF7 | 6.00 | Q9UG63 | ABCF2 | 0.14 |
| Q1KMD3 | HNRNPUL2 | 6.00 | P53621 | COPA | 0.14 |
| Q6DKK2 | TTC19 | 6.00 | P33992 | MCM5 | 0.13 |
| P31948 | STIP1 | 5.33 | O00541 | PES1 | 0.13 |
| Q9UHD8 | SEPTIN9 | 5.33 | P57740 | NUP107 | 0.13 |
|  |  |  | P78527 | PRKDC | 0.12 |
|  |  |  | Q9NYU2 | UGGT1 | 0.08 |

# Reference

# Jomini V, Oppliger-Pasquali S, Wietlisbach V, Rodondi N, Jotterand V, Paccaud F, Darioli R, Nicod P, Mooser V. [Contribution of major cardiovascular risk factor to familial premature coronary artery disease: the GENECARD project.](https://www.geenmedical.com/article?id=12204497&type=true) J. Am. Coll. Cardiol. 2002; 40(4):676-84. DOI：[10.1016/s0735-1097(02)02017-x](https://doi.org/10.1016/s0735-1097(02)02017-x)

# Marenberg ME, Risch N, Berkman LF, Floderus B, de FaireU. Genetic susceptibility to death from coronary heart disease in a study of twins. N Engl J Med. 1994 Apr 14;330(15):1041-6. doi: 10.1056/NEJM199404143301503. PMID: 8127331

# Iribarren C, Go AS, Husson G, Sidney S, Fair JM, Quertermous T, Hlatky MA, Fortmann SP. Metabolic syndrome and early-onset coronary artery disease: is the whole greater than its parts? J Am Coll Cardiol. 2006 Nov 7;48(9):1800-7. DOI: 10.1016/j.jacc.2006.03.070.PMID: 17084253

# Dallongeville J, Grupposo MC, Cottel D, Ferrières J, Arveiler D, Bingham A, Ruidavets JB, Haas B, Ducimetière P, Amouyel P. Association between the metabolic syndrome and parental history of premature cardiovascular disease. Eur. Heart J. 2006 Mar;276(6):722-8. DOI:10.1093/eurheartj/ehi717.PMID:16401673

# Rumboldt M,Rumboldt Z,Pesenti S. Association between the metabolic syndrome and parental history of premature cardiovascular disease. Eur. Heart J.2006 Oct; 27(20):2481. DOI：10.1093/eurheartj/ehl262. PMID：16984926.

# Murabito JM, Pencina MJ, Nam BH, D'Agostino RB Sr, Wang TJ, Lloyd-Jones D, Wilson PW, O'Donnell CJ.Sibling cardiovascular disease as a risk factor for cardiovascular disease in middle-aged adults. JAMA. 2005 Dec 28;294(24):3117-23. DOI: 10.1001/jama.294.24.3117.PMID: 16380592

# Lloyd-Jones DM, Nam BH, D'Agostino RB Sr, Levy D, Murabito JM, Wang TJ, Wilson PW, O'Donnell CJ. Parental cardiovascular disease as a risk factor for cardiovascular disease in middle-aged adults: a prospective study of parents and offspring.JAMA. 2004 May 12;291(18):2204-11. DOI: 10.1001/jama.291.18.2204. PMID: 15138242

# Sesso HD, Lee IM, Gaziano JM, Rexrode KM, Glynn RJ, Buring JE. Maternal and paternal history of myocardial infarction and risk of cardiovascular disease in men and women. Circulation. 2001 Jul 24;104(4):393-8. doi: 10.1161/hc2901.093115. PMID: 11468199

# Wang TJ, Nam BH, D'Agostino RB, Wolf PA, Lloyd-Jones DM, MacRae CA, Wilson PW, Polak JF, O'Donnell CJ. Carotid intima-media thickness is associated with premature parental coronary heart disease: the Framingham Heart Study. Circulation. 2003 Aug 05;108(5):572-6.DOI;[10.1161/ 01.CIR.0000081764.35431.DE](https://doi.org/10.1161/01.CIR.0000081764.35431.DE), PMID:[12874190](https://www.ncbi.nlm.nih.gov/pubmed/12874190)

# Parikh NI, Hwang SJ, Larson MG, Cupples LA, Fox CS, Manders ES, Murabito JM, Massaro JM, Hoffmann U,O'Donnell CJ. Parental occurrence of premature cardiovascular disease predicts increased coronary artery and abdominal aortic calcification in the Framingham Offspring and Third Generation cohorts. Circulation. 2007 Sep 25; 116(13):1473-81. DOI：10.1161/CIRCULATIONAHA.107.705202 .PMID：17785619

# Nasir K, Michos ED,  Rumberger JA,  Braunstein JB,  Post WS,  Budoff MJ,  Blumenthal RS.Coronary artery calcification and family history of premature coronary heart disease: sibling history is more strongly associated than parental history. Circulation.2004 Oct 12;110(15):2150-6. doi: 10.1161/01.CIR.0000144464.11080.14. PMID: 15466626

# Samani NJ,  Burton P,  Mangino M,  Ball SG,  Balmforth AJ,  Barrett J,  Bishop T,  Hall A,  BHF Family Heart Study Research Group. [A genomewide linkage study of 1,933 families affected by premature coronary artery disease: The British Heart Foundation (BHF) Family Heart Study.](https://www.geenmedical.com/article?id=16380912&type=true) Am. J. Hum. Genet. 2005 Dec; 77(6):1011-20. DOI:[10.1086/498653](https://doi.org/10.1086/498653). PMID：[16380912](https://www.ncbi.nlm.nih.gov/pubmed/16380912)

# Nielsen M, Andersson C, Gerds TA, Andersen PK, Jensen TB, Køber L, Gislason G,  Torp-Pedersen C.Familial clustering of myocardial infarction in first-degree relatives: a nationwide study. Eur Heart J. 2013 Apr;34(16):1198-203. DOI: 10.1093/eurheartj/ehs475. PMID: 23297314

# Friedlander Y, Arbogast P, Schwartz SM, Marcovina SM, Austin MA, Rosendaal FR, Reiner AP, Psaty BM, Siscovick DS.Family history as a risk factor for early onset myocardial infarction in young women.Atherosclerosis. 2001 May;156(1):201-7. doi: 10.1016/s0021-9150(00)00635-3. PMID: 11369015

# Bao W, Srinivasan SR, Valdez R, Greenlund KJ, Wattigney WA, Berenson GS. [Longitudinal changes in cardiovascular risk from childhood to young adulthood in offspring of parents with coronary artery disease: the Bogalusa Heart Study.](https://pubmed.ncbi.nlm.nih.gov/9388151/)JAMA. 1997 Dec 3;278(21):1749-54. DOI：[10.1001/jama.278.21.1749](https://doi.org/10.1001/jama.278.21.1749) PMID: 9388151

# Sdringola S, Patel D, Gould KL. High prevalence of myocardial perfusion abnormalities on positron emission tomography in asymptomatic persons with a parent or sibling with coronary artery disease. Circulation. 2001 Jan 30;103(4):496-501. doi: 10.1161/01.cir.103.4.496. PMID: 11157712

# Schächinger V, Britten MB, Elsner M, Walter DH, Scharrer I, Zeiher AM. A positive family history of premature coronary artery disease is associated with impaired endothelium-dependent coronary blood flow regulation. Circulation. 1999 Oct 5;100(14):1502-8. DOI: 10.1161/01.cir.100.14.1502.PMID: 10510052

# Gaeta G, De Michele M, Cuomo S, Guarini P, Foglia MC, Bond MG, Trevisan M.Arterial abnormalities in the offspring of patients with premature myocardial infarction.N Engl J Med. 2000 Sep 21;343(12):840-6.DOI: 10.1056/ NEJM200009213431203. PMID: 10995863

# Clarkson P, Celermajer DS, Powe AJ,Donald AE,Henry RM,Deanfield JE. Endothelium-dependent dilatation is impaired in young healthy subjects with a family history of premature coronary disease. Circulation.1997 Nov 18;96(10):3378-83. doi: 10.1161/01.cir.96.10.3378.PMID: 9396430

# De Bacquer D, De Backer G, Kornitzer M, Blackburn H. Parental history of premature coronary heart disease mortality and signs of ischemia on the resting electrocardiogram. J Am Coll Cardiol. 1999 May;33(6):1491-8. DOI: 10.1016/s0735-1097(99)00067-4. PMID: 10334413

# Mani A, Radhakrishnan J, Wang H, Mani A, Mani MA, Nelson-Williams C, Carew KS, Mane S, Najmabadi H, Wu D, Lifton RP. LRP6 mutation in a family with early coronary disease and metabolic risk factors. Science. 2007 Mar 2;315(5816):1278-82. doi: 10.1126/science.1136370. PMID: 17332414

# Keramati AR, Fathzadeh M, Go GW, Singh R, Choi M, Faramarzi S, Mane S, Kasaei M, Sarajzadeh-Fard K, Hwa J, Kidd KK, Babaee Bigi MA, Malekzadeh R, Hosseinian A, Babaei M, Lifton RP, Mani A.A form of the metabolic syndrome associated with mutations in DYRK1B. N Engl J Med.2014 May 15;370(20):1909-1919. doi: 10.1056/NEJMoa1301824. PMID: 24827035

# Xie X, Zheng YY, Adi D, Yang YN, Ma YT, Li XM, Fu ZY, Ma X, Liu F, Yu ZX, Chen Y, Huang Y.Exome Sequencing in a Family Identifies RECQL5 Mutation Resulting in Early Myocardial Infarction. Medicine (Baltimore). 2016 Feb;95(5):e2737. doi: 10.1097/MD.0000000000002737. PMID: 26844521

# .Risch N, Merikangas K.The future of genetic studies of complex human diseases. Science. 1996 Sep 13;273(5281):1516-7. DOI: 10.1126/science.273.5281.1516.PMID: 8801636

# Levy D, Ehret GB, Rice K, Verwoert GC, Launer LJ, Dehghan A, Glazer NL, Morrison AC, Johnson AD, Aspelund T, Aulchenko Y, Lumley T, Köttgen A, Vasan RS, Rivadeneira F, Eiriksdottir G, Guo X, Arking DE, Mitchell GF, Mattace-Raso FU, Smith AV, Taylor K, Scharpf RB, Hwang SJ, Sijbrands EJ, Bis J, Harris TB, Ganesh SK, O'Donnell CJ, Hofman A, Rotter JI, Coresh J, Benjamin EJ, Uitterlinden AG, Heiss G, Fox CS, Witteman JC, Boerwinkle E, Wang TJ, Gudnason V, Larson MG, Chakravarti A, Psaty BM, van Duijn CM. Genome-wide association study of blood pressure and hypertension. Nat Genet. 2009 Jun;41(6):677-87. doi: 10.1038/ng.384. PMID: 19430479

# Newton-Cheh C, Johnson T, Gateva V, Tobin MD, Bochud M, Coin L, Najjar SS, Zhao JH, Heath SC, Eyheramendy S, Papadakis K, Voight BF, Scott LJ, Zhang F, Farrall M, Tanaka T, Wallace C, Chambers JC, Khaw KT, Nilsson P, van der Harst P, Polidoro S, Grobbee DE, Onland-Moret NC, Bots ML, Wain LV, Elliott KS, Teumer A, Luan J, Lucas G, Kuusisto J, Burton PR, Hadley D, McArdle WL; Wellcome Trust Case Control Consortium, Brown M, Dominiczak A, Newhouse SJ, Samani NJ, Webster J, Zeggini E, Beckmann JS, Bergmann S, Lim N, Song K, Vollenweider P, Waeber G, Waterworth DM, Yuan X, Groop L, Orho-Melander M, Allione A, Di Gregorio A, Guarrera S, Panico S, Ricceri F, Romanazzi V, Sacerdote C, Vineis P, Barroso I, Sandhu MS, Luben RN, Crawford GJ, Jousilahti P, Perola M, Boehnke M, Bonnycastle LL, Collins FS, Jackson AU, Mohlke KL, Stringham HM, Valle TT, Willer CJ, Bergman RN, Morken MA, Döring A, Gieger C, Illig T, Meitinger T, Org E, Pfeufer A, Wichmann HE, Kathiresan S, Marrugat J, O'Donnell CJ, Schwartz SM, Siscovick DS, Subirana I, Freimer NB, Hartikainen AL, McCarthy MI, O'Reilly PF, Peltonen L, Pouta A, de Jong PE, Snieder H, van Gilst WH, Clarke R, Goel A, Hamsten A, Peden JF, Seedorf U, Syvänen AC, Tognoni G, Lakatta EG, Sanna S, Scheet P, Schlessinger D, Scuteri A, Dörr M, Ernst F, Felix SB, Homuth G, Lorbeer R, Reffelmann T, Rettig R, Völker U, Galan P, Gut IG, Hercberg S, Lathrop GM, Zelenika D, Deloukas P, Soranzo N, Williams FM, Zhai G, Salomaa V, Laakso M, Elosua R, Forouhi NG, Völzke H, Uiterwaal CS, van der Schouw YT, Numans ME, Matullo G, Navis G, Berglund G, Bingham SA, Kooner JS, Connell JM, Bandinelli S, Ferrucci L, Watkins H, Spector TD, Tuomilehto J, Altshuler D, Strachan DP, Laan M, Meneton P, Wareham NJ, Uda M, Jarvelin MR, Mooser V, Melander O, Loos RJ, Elliott P, Abecasis GR, Caulfield M, Munroe PB. Genome-wide association study identifies eight loci associated with blood pressure. Nat Genet. 2009;41(6):666-76. doi: 10.1038/ng.361. PMID: 19430483

# Schunkert H,  König IR,  Kathiresan S,  Reilly MP,  Assimes TL,  Holm H,  Preuss M,  Stewart AF,  Barbalic M,  Gieger C,  Absher D,  Aherrahrou Z,  Allayee H,  Altshuler D,  Anand SS,  Andersen K,  Anderson JL,  Ardissino D,  Ball SG,  Balmforth AJ,  Barnes TA,  Becker DM,  Becker LC,  Berger K,  Bis JC,  Boekholdt SM,  Boerwinkle E,  Braund PS,  Brown MJ,  Burnett MS,  Buysschaert I,  Cardiogenics,  Carlquist JF,  Chen L,  Cichon S,  Codd V,  Davies RW,  Dedoussis G,  Dehghan A,  Demissie S,  Devaney JM,  Diemert P,  Do R,  Doering A,  Eifert S,  Mokhtari NE,  Ellis SG,  Elosua R,  Engert JC,  Epstein SE,  deFaire U,  Fischer M,  Folsom AR,  Freyer J,  Gigante B,  Girelli D,  Gretarsdottir S,  Gudnason V,  Gulcher JR,  Halperin E,  Hammond N,  Hazen SL,  Hofman A,  Horne BD,  Illig T,  Iribarren C,  Jones GT,  Jukema JW,  Kaiser MA,  Kaplan LM,  Kastelein JJ,  Khaw KT,  Knowles JW,  Kolovou G,  Kong A,  Laaksonen R,  Lambrechts D,  Leander K,  Lettre G,  Li M,  Lieb W,  Loley C,  Lotery AJ,  Mannucci PM,  Maouche S,  Martinelli N,  McKeown PP,  Meisinger C,  Meitinger T,  Melander O,  Merlini PA,  Mooser V,  Morgan T,  Mühleisen TW,  Muhlestein JB,  Münzel T,  Musunuru K,  Nahrstaedt J,  Nelson CP,  Nöthen MM,  Olivieri O,  Patel RS,  Patterson CC,  Peters A,  Peyvandi F,  Qu L,  Quyyumi AA,  Rader DJ,  Rallidis LS,  Rice C,  Rosendaal FR,  Rubin D,  Salomaa V,  Sampietro ML,  Sandhu MS,  Schadt E,  Schäfer A,  Schillert A,  Schreiber S,  Schrezenmeir J,  Schwartz SM,  Siscovick DS,  Sivananthan M,  Sivapalaratnam S,  Smith A,  Smith TB,  Snoep JD,  Soranzo N,  Spertus JA,  Stark K,  Stirrups K,  Stoll M,  Tang WH,  Tennstedt S,  Thorgeirsson G,  Thorleifsson G,  Tomaszewski M,  Uitterlinden AG,  van Rij AM,  Voight BF,  Wareham NJ,  Wells GA,  Wichmann HE,  Wild PS,  Willenborg C,  Witteman JC,  Wright BJ,  Ye S,  Zeller T,  Ziegler A,  Cambien F,  Goodall AH,  Cupples LA,  Quertermous T,  März W,  Hengstenberg C,  Blankenberg S,  Ouwehand WH,  Hall AS,  Deloukas P,  Thompson JR,  Stefansson K,  Roberts R,  Thorsteinsdottir U,  O'Donnell CJ,  McPherson R,  Erdmann J,  CARDIoGRAM Consortium,  Samani NJ. [Large-scale association analysis identifies 13 new susceptibility loci for coronaryartery disease.](https://www.geenmedical.com/article?id=21378990&type=true)Nat. Genet. 2011 Mar 06;  43(4):333-8. DOI：[10.1038/ng.784](https://doi.org/10.1038/ng.784)  .PMID: 21378990

# Lu X, Wang L, Lin X, Huang J, Charles Gu C, He M, Shen H, He J, Zhu J, Li H, Hixson JE, Wu T, Dai J, Lu L, Shen C, Chen S, He L, Mo Z, Hao Y, Mo X, Yang X, Li J, Cao J, Chen J, Fan Z, Li Y, Zhao L, Li H, Lu F, Yao C, Yu L, Xu L, Mu J, Wu X, Deng Y, Hu D, Zhang W, Ji X, Guo D, Guo Z, Zhou Z, Yang Z, Wang R, Yang J, Zhou X, Yan W, Sun N, Gao P, Gu D. Genome-wide association study in Chinese identifies novel loci for blood pressure and hypertension.Hum Mol Genet. 2015 Feb 1;24(3):865-74. doi: 10.1093/hmg/ddu478. Epub 2014 Sep 23.PMID: 25249183

# Wang Y, Wang L, Liu X, Zhang Y, Yu L, Zhang F, Liu L, Cai J, Yang X, Wang X. Genetic variants associated with myocardial infarction and the risk factors in Chinese population. PLoS One. 2014 Jan 27;9(1):e86332. doi: 10.1371/journal.pone.0086332. eCollection 2014. PMID: 24475106

# Xi B, Shen Y, Zhao X, Chandak GR, Cheng H, Hou D, Li Y, Ott J, Zhang Y, Wang X, Mi J.Association of common variants in/near six genes (ATP2B1, CSK, MTHFR, CYP17A1, STK39 and FGF5) with blood pressure/hypertension risk in Chinese children. J Hum Hypertens. 2014 Jan;28(1):32-6. doi: 10.1038/jhh.2013.50. Epub 2013 Jun 13. PMID: 23759979

# Diver LA, MacKenzie SM, Fraser R, McManus F, Freel EM, Alvarez-Madrazo S, McClure JD, Friel EC, Hanley NA, Dominiczak AF, Caulfield MJ, Munroe PB, Connell JM, Davies E.Common Polymorphisms at the CYP17A1 Locus Associate With Steroid Phenotype: Support for Blood Pressure Genome-Wide Association Study Signals at This Locus. Hypertension. 2016 Apr;67(4):724-732. doi: 10.1161/HYPERTENSIONAHA.115.06925. Epub 2016 Feb 22. PMID: 26902494

# López-Mejías R, Corrales A, Vicente E, Robustillo-Villarino M, González-Juanatey C, Llorca J, Genre F, Remuzgo-Martínez S, Dierssen-Sotos T, Miranda-Filloy JA, Huaranga MA, Pina T, Blanco R, Alegre-Sancho JJ, Raya E, Mijares V, Ubilla B, Ferraz-Amaro I, Gómez-Vaquero C, Balsa A, López-Longo FJ, Carreira P, González-Álvaro I, Ocejo-Vinyals JG, Rodríguez-Rodríguez L, Fernández-Gutiérrez B, Castañeda S, Martín J, González-Gay MA. Influence of coronary artery disease and subclinical atherosclerosis related polymorphisms on the risk of atherosclerosis in rheumatoid arthritis. Sci Rep. 2017 Jan 6;7:40303. doi: 10.1038/srep40303. PMID: 28059143

# Dai CF, Xie X, Ma YT, Yang YN, Li XM, Fu ZY, Liu F, Chen BD, Gai MT. Haplotype analyses of CYP17A1 genetic polymorphisms and coronary artery disease in a Uygur population.J Renin Angiotensin Aldosterone Syst. 2015 Jun;16(2):389-98. doi: 10.1177/1470320314565840. Epub 2015 Jan 14. PMID: 25592814

# Dai CF, Xie X, Yang YN, Li XM, Zheng YY, Fu ZY, Liu F, Chen BD, Gai MT, Ma YT. Relationship between CYP17A1 genetic polymorphism and coronary artery disease in a Chinese Han population. Lipids Health Dis. 2015 Mar 7; 14:16. doi: 10.1186/s12944-015-0007-4. PMID: 25889125

# Aherrahrou R, Kulle AE, Alenina N, Werner R, Vens-Cappell S, Bader M,Schunkert H, Erdmann J, Aherrahrou Z. CYP17A1 deficient XY mice display susceptibility to atherosclerosis, altered lipidomic profile and atypical sex development. Sci Rep. 2020 05 29; 10(1):8792. doi: 10.1038/s41598-020-65601-0.PMID: 32472014

# Werner H, Sarfstein R, Laron Z. The Role of Nuclear Insulin and IGF1 Receptors in Metabolism and Cancer. Biomolecules. 2021 Apr 2;11(4):531. doi: 10.3390/biom11040531. PMID: 33918477; PMCID: PMC8065599.

# Higashi Y, Gautam S, Delafontaine P, Sukhanov S. IGF-1 and cardiovascular disease. Growth Horm IGF Res. 2019 Apr;45:6-16. doi: 10.1016/j.ghir.2019.01.002. Epub 2019 Jan 31. PMID: 30735831; PMCID: PMC6504961.

# Forbes BE, Blyth AJ, Wit JM. Disorders of IGFs and IGF-1R signaling pathways. Mol Cell Endocrinol. 2020 Dec 1;518:111035. doi: 10.1016/j.mce.2020.111035. Epub 2020 Sep 15. PMID: 32941924.

# Zhang Z, Yao L, Yang J, Wang Z, Du G. PI3K/Akt and HIF‑1 signaling pathway in hypoxia‑ischemia (Review). Mol Med Rep. 2018 Oct;18(4):3547-3554. doi: 10.3892/mmr.2018.9375. Epub 2018 Aug 9. PMID: 30106145; PMCID: PMC6131612.

# Ancey PB, Contat C, Meylan E. Glucose transporters in cancer - from tumor cells to the tumor microenvironment. FEBS J. 2018 Aug;285(16):2926-2943. doi: 10.1111/febs.14577. Epub 2018 Jun 25. PMID: 29893496.

# Chow CK, Pell AC, Walker A, O'Dowd C, Dominiczak AF, Pell JP.Families of patients with premature coronary heart disease: an obvious but neglected target for primary prevention. BMJ. 2007 Sep 8;335(7618):481-5. doi: 10.1136/bmj.39253.577859.BE. PMID: 17823190

# Mulders TA, Sivapalaratnam S,Stroes ES,Kastelein JJ,Guerci AD,Pinto-Sietsma SJ.Asymptomatic individuals with a positive family history for premature coronary artery disease and elevated coronary calcium scores benefit from statin treatment: a post hoc analysis from the St. Francis Heart Study.JACC Cardiovasc Imaging. 2012 Mar;5(3):252-60.DOI:10.1016/j.jcmg.2011.11.014. PMID: 22421169

# Mega JL, Stitziel NO, Smith JG, Chasman DI, Caulfield M, Devlin JJ, Nordio F, Hyde C, Cannon CP, Sacks F, Poulter N, Sever P, Ridker PM, Braunwald E, Melander O, Kathiresan S, Sabatine MS.Genetic risk, coronary heart disease events, and the clinical benefit of statin therapy: an analysis of primary and secondary prevention trials.Lancet. 2015 Jun 6;385(9984):2264-2271. doi: 10.1016/S0140-6736(14)61730-X. PMID: 25748612

# O'Donnell CJ, Larson MG, Feng D, Sutherland PA, Lindpaintner K, Myers RH, D'Agostino RA, Levy D, Tofler GH; Framingham Heart Study. Genetic and environmental contributions to platelet aggregation: the Framingham heart study.Circulation. 2001 Jun 26;103(25):3051-6. doi: 10.1161/01.cir.103.25.3051. PMID: 11425767

# Magadle R, Merlon H, Weiner P, Mohammedi I, Robert D.C-reactive protein levels and arterial abnormalities in the offspring of patients with premature myocardial infarction. Cardiology. 2003;100(1):1-6. doi: 10.1159/000072384. PMID: 12975538

# Auchus RJ.The genetics, pathophysiology, and management of human deficiencies of P450c17.Endocrinol Metab Clin North Am. 2001 Mar;30(1):101-19, vii. doi: 10.1016/s0889-8529(08)70021-5. PMID: 11344930

# Crandall CJ, Barrett-Connor E.Endogenous sex steroid levels and cardiovascular disease in relation to the menopause: a systematic review.Endocrinol Metab Clin North Am. 2013 Jun;42(2):227-53.doi: 10.1016/j.ecl.2013.02.003.PMID: 23702399

# Zhao D, Guallar E, Ouyang P, Subramanya V, Vaidya D, Ndumele CE, Lima JA, Allison MA, Shah SJ, Bertoni AG, Budoff MJ, Post WS, Michos ED.Endogenous Sex Hormones and Incident Cardiovascular Disease in Post-Menopausal Women.J Am Coll Cardiol. 2018 Jun 5;71(22):2555-2566. doi: 10.1016/j.jacc.2018.01.083.PMID: 29852978

# Rallidis LS, Kotakos C, Tsalavoutas S, Katsimardos A, Drosatos A, Rallidi M, Moustaka E, Zolindaki M. Low Serum Free Testosterone Association With Cardiovascular Mortality in Men With Stable CAD.J Am Coll Cardiol. 2018 Nov 27;72(21):2674-2675.doi: 10.1016/j.jacc.2018.08.2189.PMID: 30466526

# Bianchi VE.Testosterone, myocardial function, and mortality.Heart Fail Rev. 2018 Sep;23(5):773-788. doi: 10.1007/s10741-018-9721-0.PMID: 29978359

# Adelborg K, Rasmussen TB, Nørrelund H, Layton JB, Sørensen HT, Christiansen CF.Cardiovascular Outcomes and All-cause Mortality Following Measurement of Endogenous Testosterone Levels.Am J Cardiol. 2019 Jun 1;123(11):1757-1764.doi: 10.1016/j.amjcard.2019.02.042. Epub 2019 Mar 8. PMID: 30928032

# Wu TT, Gao Y, Zheng YY, Ma YT, Xie X.Association of endogenous DHEA/DHEAS with coronary heart disease: A systematic review and meta-analysis.Clin Exp Pharmacol Physiol. 2019 Nov;46(11):984-994. doi: 10.1111/1440-1681.13146.PMID: 31347187

# Nakajin S, Shively JE, Yuan PM, Hall PF.Microsomal cytochrome P-450 from neonatal pig testis: two enzymatic activities (17 alpha-hydroxylase and c17,20-lyase) associated with one protein.Biochemistry.1981 Jul 7;20(14):4037-42. doi: 10.1021/bi00517a014.PMID: 6793062

# Lu Y, Wang E, Chen Y, Zhou B, Zhao J, Xiang L, Qian Y,Jiang J, Zhao L, Xiong X, Lu Z, Wu D, Liu B, Yan J, Zhang R, Zhang H, Hu C, Li X. Obesity-induced excess of 17-hydroxyprogesterone promotes hyperglycemia through activation of glucocorticoid receptor. The Journal of clinical investigation. 2020 Jul 01; 130(7):3791-3804. doi: 10.1172/JCI134485.PMID: 32510471

# Villablanca A, Lubahn D,Shelby L,Lloyd K,Barthold S.Susceptibility to early atherosclerosis in male mice is mediated by estrogen receptor alpha.Arterioscler Thromb Vasc Biol.2004 Jun;24(6):1055-61. doi:10.1161/01.ATV.0000130467.65290.d4. PMID: 15117737

# Villablanca AC,Tenwolde A,Lee M,Huck M, Mumenthaler S,Rutledge JC.17beta-estradiol prevents early-stage atherosclerosis in estrogen receptor-alpha deficient female mice.J Cardiovasc Transl Res .2009 Sep;2(3):289-99.doi: 10.1007/s12265-009-9103-z.PMID: 19654889
